# PLX5622 Reduces Disease Severity in Lethal CNS Infection by Off-Target Inhibition of Peripheral Inflammatory Monocyte Production

**DOI:** 10.3389/fimmu.2022.851556

**Published:** 2022-03-25

**Authors:** Alanna G. Spiteri, Duan Ni, Zheng Lung Ling, Laurence Macia, Iain L. Campbell, Markus J. Hofer, Nicholas J. C. King

**Affiliations:** ^1^ Viral Immunopathology Laboratory, Infection, Immunity and Inflammation Research Theme, School of Medical Sciences, Faculty of Medicine and Health, The University of Sydney, Sydney, NSW, Australia; ^2^ Sydney Cytometry, The University of Sydney and Centenary Institute, Sydney, NSW, Australia; ^3^ Ramaciotti Facility for Human Systems Biology, The University of Sydney and Centenary Institute, Sydney, NSW, Australia; ^4^ Charles Perkins Centre, The University of Sydney, Sydney, NSW, Australia; ^5^ Chronic Diseases Research Theme, School of Medical Sciences, Faculty of Medicine and Health, The University of Sydney, Sydney, NSW, Australia; ^6^ School of Life and Environmental Sciences, The University of Sydney, Sydney, NSW, Australia; ^7^ The University of Sydney Institute for Infectious Diseases, The University of Sydney, Sydney, NSW, Australia; ^8^ The University of Sydney Nano Institute, The University of Sydney, Sydney, NSW, Australia

**Keywords:** microglia, monocyte-derived cells, neuroinflammation, West Nile virus-induced encephalitis, CSF-1R antagonism, microglia depletion, CNS infection, monocyte-mediated inflammation

## Abstract

PLX5622 is a CSF-1R inhibitor and microglia-depleting reagent, widely used to investigate the biology of this central nervous system (CNS)-resident myeloid population, but the indirect or off-target effects of this agent remain largely unexplored. In a murine model of severe neuroinflammation induced by West Nile virus encephalitis (WNE), we showed PLX5622 efficiently depleted both microglia and a sub-population of border-associated macrophages in the CNS. However, PLX5622 also significantly depleted mature Ly6C^hi^ monocytes in the bone marrow (BM), inhibiting their proliferation and lethal recruitment into the infected brain, reducing neuroinflammation and clinical disease scores. Notably, in addition, BM dendritic cell subsets, plasmacytoid DC and classical DC, were depleted differentially in infected and uninfected mice. Confirming its protective effect in WNE, cessation of PLX5622 treatment exacerbated disease scores and was associated with robust repopulation of microglia, rebound BM monopoiesis and markedly increased inflammatory monocyte infiltration into the CNS. Monoclonal anti-CSF-1R antibody blockade late in WNE also impeded BM monocyte proliferation and recruitment to the brain, suggesting that the protective effect of PLX5622 is *via* the inhibition of CSF-1R, rather than other kinase targets. Importantly, BrdU incorporation in PLX5622-treated mice, suggest remaining microglia proliferate independently of CSF-1 in WNE. Our study uncovers significantly broader effects of PLX5622 on the myeloid lineage beyond microglia depletion, advising caution in the interpretation of PLX5622 data as microglia-specific. However, this work also strikingly demonstrates the unexpected therapeutic potential of this molecule in CNS viral infection, as well as other monocyte-mediated diseases.

## 1 Introduction

Monocytes are derived from hematopoietic stem and progenitor cells in the adult bone marrow (BM) in the process of monopoiesis (1). Signaling *via* the colony-stimulating factor-1 receptor R (CSF-1R, CD115), a homodimeric glycoprotein, is required for the survival, differentiation, and proliferation of these cells (2–5). During “emergency” conditions, classical Ly6C^hi^ monocytes migrate from the BM *via* CCL2-CCR2 signaling into inflamed tissues (6, 7). Acute West Nile virus (WNV) infection of the central nervous system (CNS) parenchyma results in the lethal recruitment of BM-derived classical Ly6C^hi^ monocytes into the CNS (6, 8–12). These monocyte-derived cells (MCs) often adopt an immunophenotype similar to microglia, the resident parenchymal myeloid cells of the CNS, confounding clear discrimination between these cells during infection, making it difficult to accurately ascribe disease response functions to each (12–14).

In the United States, WNV is the third leading cause of all virus-induced hospitalized encephalitis (15). Since its introduction into the United States in 1999 in a lethal outbreak, it has spread throughout the American continent to occupy the largest world-wide distribution among *Flaviviridae* (16–18). Neuroinvasion, the most debilitating feature of WNV, occurs in approximately 1% of cases, resulting in meningitis, encephalitis and/or acute flaccid paralysis, with 8-10% fatality (15, 17, 19–21). This neurotropism (18) and the tendency to cause immunopathology (22–24), along with its expanding distribution and propensity to cause epidemics (25) that are unpredictable in location and magnitude, make WNV a continuing global threat (15). While an effective vaccine remains a key goal for disease control, understanding the pathogenesis of disease caused by infection remains crucial to the development of targeted therapeutic approaches to ameliorate viral immunopathology.

Tools to study the contribution of resident and infiltrating myeloid cells to the pathogenesis of CNS virus infection have, until recently, been limited. Prior to the commercial availability of *microglia-specific* reagents, few studies examined the role of microglia *in vivo*, instead utilising *in vitro* or *ex vivo* slice culture models (26–28). However, our growing understanding of the inextricable importance of the brain microenvironment in instructing myeloid phenotype and function, has driven an increasing emphasis on work *in vivo* (29, 30). *In vivo* investigation of these cells has been substantively aided by the small molecule inhibitor of CSF-1R tyrosine kinase activity, PLX5622, typically formulated into a standard rodent chow to enable oral administration. This drug readily penetrates the blood brain barrier (BBB) and can deplete > 90% of microglia in as little as three days (31–33). Removal of PLX5622 causes rapid microglia replenishment *via* the proliferation of surviving microglia (34). PLX5622 has a 20-fold higher selectivity for CSF-1R over other similar receptor tyrosine kinases, including KIT and fms-like tyrosine kinase 3 (FLT3), as well as an increased BBB penetration, making it more efficacious and specific than previously developed CSF-1R inhibitors (33). The availability of PLX5622 has thus stimulated a surge of experiments examining the role of microglia in viral encephalitis. In murine models of viral encephalitis caused by WNV, Japanese encephalitis virus, Theiler’s encephalomyelitis virus (TMEV), pseudorabies virus (PRV), Herpes Simplex virus, the neurotropic JHM strain of mouse hepatitis virus (JHMV) and Murine Hepatitis Virus (MHV), PLX5622-mediated microglia depletion resulted in increased mortality, with increased viral loads in the CNS, implying a protective role for microglia in viral infection (31, 35–42).

PLX5622 has largely been assumed to be microglia-specific, with few studies considering indirect or off-target effects produced by this molecule in the interpretation of experimental findings. However, other cells besides microglia express CSF-1R (43) and rely on its signaling for survival and proliferation (2–5). Not surprisingly, studies have demonstrated effects on peripheral myeloid and lymphoid cells post-treatment with PLX5622 (44). Non-parenchymal brain-resident border-associated macrophages (BAMs) are also likely affected by CSF-1R inhibition. Thus, identifying the role of microglia may be impossible using this molecule alone, with off-target effects potentially contributing to the disease phenotype (14).

In contrast to previously published work using PLX5622 to investigate microglial responses in WNV encephalitis (WNE), we used a 100% lethal dose (LD_100_) of WNV, inoculated intranasally to study the response of the brain independently of the systemic immune response (6). Inoculated intranasally, WNV directly infects the CNS *via* the olfactory nerve without disrupting the BBB (6). In this model, the recruitment of peripherally-derived MCs into the CNS ultimately drives immunopathology and mortality, since inhibiting their recruitment to the brain can ameliorate disease and increase survival (6, 8, 10, 11). Thus, here we investigated the impact of PLX5622-induced microglia depletion on a monocyte-mediated inflammatory disease.

In contrast to other models of viral encephalitis, we found that PLX5622 treatment prior to WNV-infection was protective. PLX5622 reduced the number and proliferative capacity of mature monocytes in the BM, resulting in a substantial decrease in the immigration of inflammatory macrophages into the CNS, as well as a corresponding reduction in cytokine expression. Strikingly, cessation of PLX5622 treatment reversed this protective effect, resulting in rebound monopoiesis and enhanced CNS infiltration, revealing CSF1-R as a potential therapeutic target. Consistent with this, monoclonal anti-CSF-1R antibody blockade late in disease reduced BM monocyte production and infiltration of these cells into the inflamed CNS, suggesting that the protective effect of PLX5622 is *via* the inhibition of CSF-1R, rather than other kinase targets. Thus, while advising caution in the interpretation of experiments in which PLX5622 has been assumed to be microglia-specific, this study demonstrates for the first time the protective effect of PLX5622 in CNS viral infection, as well as other monocyte-mediated diseases, providing new insight into the importance of CSF-1-CSF-1R signaling in monopoiesis and the recruitment of inflammatory monocytes into inflamed tissues.

## 2 Materials and Methods

### 2.1 Mice

Female 9-10-week-old C57BL/6 mice from the Animal Resource Centre (ARC) (Western Australia, Australia) were kept in individually ventilated cages under specific pathogen-free conditions with access to food and water ad libitum. All experiments were performed in accordance with National Health and Medical Research Council’s ethical guidelines with the animal ethics approval number 2019/1696 approved by the University of Sydney Animal Ethics Committee.

### 2.2 WNV Infection

Mice were anesthetised with isoflurane prior to being infected intranasally with WNV (Sarafend, a lineage II strain of WNV) delivered in 10 μL of sterile PBS [as previously described (6, 45)]. Mice were infected with 1.2 x 10^5^ plaque forming units (PFU) or 7 x 10^3^ PFU of WNV, doses that are lethal in 100% and 50% of mice, respectively. Mice were sacrificed no later than days post infection (dpi) 7. Diseases scores were recorded based on the criteria shown in **Supplementary Table 1**.

### 2.3 PLX5622-Mediated Microglia Depletion

Plexxikon Inc. (USA) provided the PLX5622 which was formulated in AIN-76A standard chow by Research diets (USA) (1200 ppm). All mice were feed PLX5622 or control chow (AIN-76A) for 21 days prior to infection. Mice were fed either PLX5622 or AIN-76A for no longer than an additional 7 days post infection.

### 2.4 Modulating Entry of Monocytes Into the CNS

#### 2.4.1 Intravenous Delivery of Clodronate Liposomes

Clodronate liposomes (Liposoma, AMS) were vortexed and delivered intravenously *via* the lateral tail vein at dpi 5 at a dose of 200 μL.

#### 2.4.2 Intraperitoneal Delivery of Blocking Antibodies

Monoclonal blocking antibodies, anti-Ly6C (BE0203) and anti-CSF-1R (CD115, AFS98) and their isotype control (2A3) (BioXcell, USA) were injected interperitoneally at either 1) dpi 5 and 6 (anti-Ly6C and anti-CSF-1R) or 2) dpi 0, 2, 4 and 6 (anti-CSF-1R) at a dose of 200 μg prepared in 200 μl of sterile PBS.

### 2.5 Tracking Recently Infiltrating Cells in the CNS Using PKH67

As per the manufacturer’s instructions, PKH26 cell-linker was mixed with diluent C (Sigma-Aldrich, USA) prior to use. PKH26 cell-linker was used at a 10-fold higher concentration than recommended and injected intravenously *via* the lateral tail vein 2 hrs prior to tissue collection.

### 2.6 Detection and Quantification of Proliferating Cells With BrdU

Mice were injected intraperitoneally with 1 mg of bromodeoxyuridine (BrdU) (Sigma-Aldrich, USA) in 200 µL sterile PBS 3 hrs before sacrifice.

### 2.7 Plaque Assay to Determine Viral Titer in the CNS

A virus plaque assay was performed as previously described (6) using virally susceptible BHK cells. Brain tissue was dissociated with a power homogenizer (TissueLyser, Qiagen) before being clarified by centrifugation. Tenfold dilutions of tissue homogenates were used to infect BHK cells. The inoculum was removed after a 1 hr incubation and replaced with an Agarose plug. Cells were incubated for a further 3 days before being fixed with 10% formalin (Sigma-Aldrich, USA). A 3% crystal violet (Sigma-Aldrich, USA) dye solution in 20% methanol (Fronine) was used to stain fixed cells. The PFU per gram was determined by factoring the number of plaques, the inoculum volume, and the dilution.

### 2.8 RNA Extraction and Real-Time Quantitative Polymerase Chain Reaction

For RNA extraction, brain tissue was dissociated with TRI Reagent (Sigma Aldrich, USA) using a tissue homogenizer (TissueLyser, Qiagen, DE). The High-Capacity cDNA Reverse Transcription Kit (ThermoFisher Scientific, USA) was used to generate cDNA and the Power SYBR™ Green PCR Master Mix (ThermoFisher Scientific, USA) was used to conduct qPCR using primers all purchased from Sigma Aldrich, USA (See **Supplementary Table 2**), on the LightCycle^®^ 480 Instrument II (Roche, CH). Gene expression values were normalized to *Rpl13a*.

### 2.9 Flow Cytometry

Prior to collection of spleen, brain and femurs, mice were anaesthetised and transcardially perfused with ice cold sterile PBS. Femurs were flushed with cold PBS using a 30-gauge needle, while spleens were gently mashed through a 70 μM nylon mesh sieve using a syringe plunger. RBC lysis buffer (Invitrogen, USA) was used to lyse erythrocytes in single cell suspensions of BM cells and splenocytes. Brains were processed into single cell suspensions in PBS and DNase I (DN25, 0.05 mg/mL) and collagenase (5 mg/mL) (Sigma-Aldrich, USA) using the gentleMACS dissociator (Miltenyi Biotec, DE). Subsequently, a 30%/80%Percoll gradient was used to isolate the cells from brain homogenates. After tissue processing, live cells were counted with trypan blue (0.4%) on a haemocytometer. Single cell suspensions were incubated with purified anti-CD16/32 (Biolegend, USA) and UV-excitable LIVE/DEAD Blue (UVLD) (Invitrogen, USA) or Zombie UV Fixable Viability kit (Biolegend, USA) and subsequently stained with a cocktail of fluorescently-labelled antibodies (See **Supplementary Table 3**). Cells were washed two times and fixed in fixation buffer (Biolegend). Intracellular antibodies were stained after surface staining, fixation and incubation with Cytofix/Cytoperm (BD Biosciences, USA). Anti-BrdU (3D4 or Bu20a, Biolegend, USA) was stained intranuclearly, as previously described (46). Briefly, after cell surface staining and fixation, cells were incubated in Cytofix/Cytoperm (BD Biosciences, USA), Cytoperm Permeabilization Buffer Plus (BD Biosciences, USA) and DNase (DN25, 30 U/sample) (Sigma-Aldrich, USA), prior to being stained with anti-BrdU.

Fluorescently-tagged antibodies were measured on the 5-laser Aurora, Spectral cytometer (Cytek Biosciences, USA). Acquired data was analysed in FlowJo (v10.8, BD Biosciences, USA). Quality control gating including time, single cells, non-debris/cells and Live/Dead staining was applied to exclude debris, doublets and dead cells. Cell numbers were quantified using cell proportions exported from FlowJo and total live cell counts.

### 2.10 tSNE Analysis

The FCS files were compensated and gated in FlowJo prior to exporting channel values (CSV). T-distributed stochastic neighbour embedding (tSNE) was applied to CSV files, in RStudio (1.1.453 or 1.4.1717) using CAPX (46) (package publicly available: https://github.com/sydneycytometry/CAPX) or Spectre (47) (package publicly available: https://github.com/ImmuneDynamics/Spectreusing default settings) using default settings i.e., perplexity = 30, theta = 0.5 and iterations = 1000.

### 2.11 Heatmaps

The FCS files were compensated and gated in FlowJo prior to exporting median fluorescent intensity (MFI) signals from populations of interest. Heatmaps were applied to MFI’s in RStudio (1.2.1335) using the R package, *pheatmaps* (48).

### 2.12 Statistical Analysis

Non-parametric statistical tests were applied to data in GraphPad Prism 8.4.3 (GraphPad Software, La Jolla, CA). Comparison of two groups was conducted using Mann–Whitney test, and three or more groups were compared using a Kruskal–Wallis test with a Dunn’s multiple comparison test. When two independent variables and three or more groups were being compared a Two-way ANOVA and a Šídák’s or Tukey’s multiple comparisons test was used. Error bars are shown as standard error of the mean (SEM).

## 3 Results

### 3.1 PLX5622-Mediated Microglia Depletion Is Protective in WNE and Reversed Upon Cessation of Treatment

**Figure 1:**
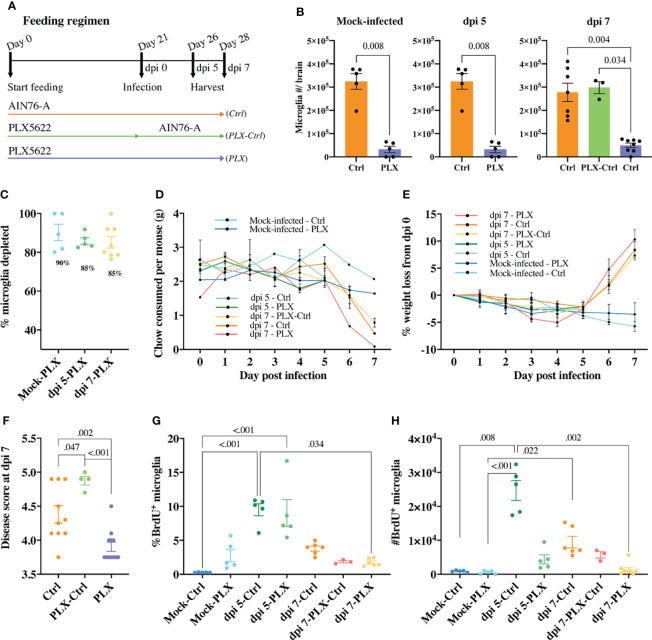
PLX5622-mediated microglia depletion is protective in WNE and reversed upon cessation of treatment. **(A)** Schematic of experimental design. Mice were fed control chow (AIN-76A, Ctrl) or PLX5622-formulated chow until sacrifice at dpi 5 (or day 26) or dpi 7 (or day 28) (*PLX*) or fed PLX5622-formulated chow until dpi 0 (or day 21) and then fed control chow (AIN-76A) until dpi 7 (*PLX-Ctrl*). **(B)** Number of microglia in the brain of mock-infected and infected *Ctrl* and *PLX* mice at dpi 5 and 7 and infected *PLX-Ctrl* mice at dpi 7. **(C)** Percent of microglia depleted in PLX5622-treated and mock-infected or infected mice at dpi 5 and 7. **(D, E)** Weight (g) of chow consumed **(D)** and percent of weight lost **(E)** by mock-infected and infected mice culled at dpi 5 or 7. **(F)** Disease score of *Ctrl*, *PLX-Ctrl* and *PLX* mice at WNV dpi 7. **(G, H)** Number and percent of BrdU^+^ microglia in mock-infected and infected *Ctrl* and *PLX* mice at dpi 5 and 7 and infected *PLX-Ctrl* mice at dpi 7. Data is presented as mean ± SEM from two independent experiments with at least three mice per group.

The depletion of microglia using PLX5622 provides a non-invasive approach to study the *in vivo* functions of microglia during disease. Previously published studies show that microglia depletion exacerbates clinical scores and mortality in murine models of viral CNS infection (31, 36–40). To investigate this in more detail, we set up 3 principal groups, as follows. In the experimental group *(PLX)*, we fed mice PLX5622-formulated chow for 21 days prior to infection and following this, for 5 or 7 dpi, to induce and maintain maximal microglia depletion. In the second group *(PLX-Ctrl)*, after 21 days we replaced PLX5622-formulated chow with control chow (AIN-76A) from dpi 0 (the day of infection) onwards, to enable the replenishment of microglial numbers *via* proliferation of surviving microglia (34). In the master control group *(Ctrl)*, we fed mice only control chow (AIN-76A) throughout the period until sacrifice at dpi 5 or 7 (**Figure 1A**).

In the mock-infected *PLX* group, 90% of microglia were depleted (**Figures 1B, C**). We also observed a significant decrease in the number of CD45^+^, Ly6G^-^, CD3e^-^, NK1.1^-^, Ly6C^-^, CD11b^+^, CD68^+^, MHC-II^+^ cells, a phenotype consistent with a subpopulation of border-associated macrophages (BAMs) (**Supplementary Figure 1**). Further, in the brains of this group, we saw an unexpected increase in the number of neutrophils, T cells and NK cells, possibly recruited in response to dead microglia in the CNS (**Supplementary Figure 1**). In *PLX* mice, a mean depletion of 85% microglia was evident in WNV-infected mice at dpi 5. Depletion remained at 85% on dpi 7, despite reduced chow consumption by infected animals on dpi 6 and 7 (**Figures 1B–D**). Strikingly, however, the WNV-infected *PLX* group showed a significantly lower clinical disease score at dpi 7 than the *Ctrl* group (**Figure 1F**). In contrast, in the *PLX-Ctrl* group, substitution of PLX5622 chow with control chow on dpi 0 of infection was associated with restoration of microglial numbers and a significant increase in disease score by dpi 7 (**Figures 1B, F**). Despite differences in disease score, all infected groups showed a similar degree of weight loss from dpi 5 onwards, losing >5% of their initial weight by dpi 7 (**Figure 1E**).

Since microglia are putatively protective during viral encephalitis (31, 36–40), we investigated whether the decrease in disease score in the *PLX* group resulted from microglial repopulation, with the decreased chow consumed by mice late in infection. The number and percent of proliferating microglia in the *PLX* group, measured using BrdU incorporation *in vivo*, was not significantly different from *Ctrl* or *PLX-Ctrl* groups at dpi 7 (**Figures 1G, H**). Thus, it is unlikely that the amelioration of disease scores conferred by PLX5622 was due to microglial proliferation, especially as disease scores in the *Ctrl* group were re-capitulated, if not exceeded, in the microglia-repopulated *PLX-Ctrl* group. Intriguingly, however, in both mock-infected and infected PLX5622-treated mice, microglia were still evidently proliferating (**Figures 1G, H**). At dpi 5, when peak microglia proliferation occurs in WNE (12), PLX5622 did not inhibit the proliferative capacity of remaining microglia, as the frequency of BrdU^+^ microglia (~9%) was no different from the *Ctrl* group (**Figure 1G**). Overall, these data suggest that PLX5622 is protective during lethal viral infection and that a CSF-1-independent mechanism of microglial proliferation during CSF1-R blockade becomes more conspicuous in WNE.

### 3.2 Correlation of Viral Load and Microglia Number in WNV-Infection in Microglia-Depleted Brains

**Figure 2:**
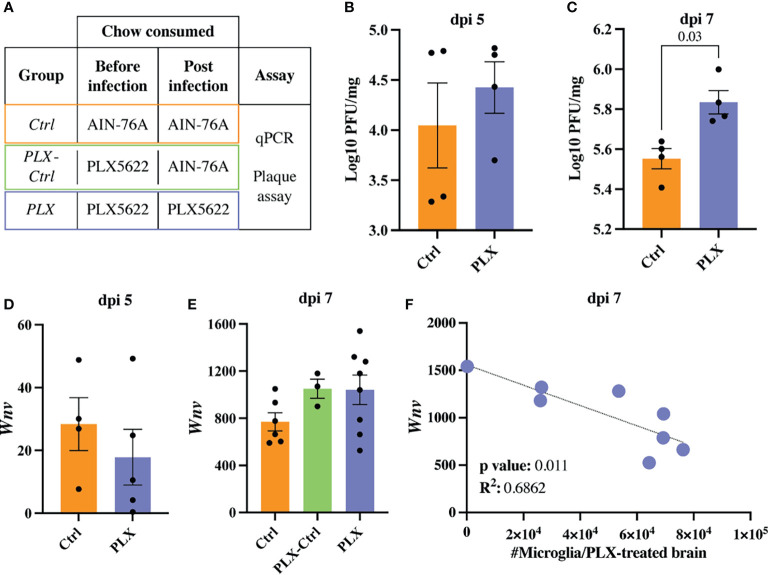
Increasing numbers of microglia are required to reduce viral load late in WNV-infection. **(A)** Table showing experimental groups. **(B, C)** PFU of WNV in brains of *PLX* and *Ctrl* mice at dpi 5 **(B)** and 7 **(C)**. **(D, E)** Expression of *Wnv* as determined by qPCR in brains of infected *Ctrl* and *PLX* mice at dpi 5 **(D)** and 7 **(E)** and *PLX-Ctrl* at dpi 7 **(E)**. *Wnv* was normalized to the housekeeping gene, *Rpl13a.* **(F)** Correlation analysis between the expression of *Wnv* and the number of microglia in *PLX* mice at dpi 7. Data is presented as mean ± SEM from one (plaque assay) or two (qPCR data) independent experiments with at least three mice per group.

To investigate the protective effect of PLX5622 during WNV-infection, we assessed viral loads in the brain at dpi 5 and 7 using a viral plaque assay and q-PCR (**Figures 2A–E**). Compared to *Ctrl* mice, plaque assays on homogenized brains from *PLX* mice showed no change at dpi 5 and slight increase in PFU at dpi 7 (**Figures 2B, C**). Using q-PCR, we observed no significant differences at either timepoint (**Figures 2D, E**). However, there was an increase in viral RNA at dpi 7 (albeit non-significant) and a significant inverse correlation between viral RNA and numbers of microglia in the brain in *PLX* mice at dpi 7 (**Figures 2E, F**), supporting the association of microglia with viral clearance (31, 35, 36, 38). Interestingly, microglial restoration in *PLX-Ctrl* mice showed no difference in viral load *via* qPCR, compared to microglia-depleted *PLX* mice (**Figure 2E**). While a plaque assay was not performed on this group to confirm these findings, this could suggest that repopulated microglia are less capable of clearing virus. Supporting this, P2RY12, a nominal microglia-enriched (29) purinergic receptor was reduced by more than 50% on repopulating microglia in the *PLX-Ctrl* group and remaining microglia in the *PLX* group at dpi 7, relative to the *Ctrl* group (**Supplementary Figure 2**). This molecule is associated with an enhanced microglial migratory capacity and ability to phagocytose infected neurons for viral clearance (35), thus a reduction in P2RY12 may have inhibited viral clearance by repopulated microglia. Nonetheless, the increased viral titres in the *PLX* group do not explain the protective effect of PLX5622 in WNV-infection.

### 3.3 Treatment With PLX5622 Reduces Leukocyte Influx and the Neuroinflammatory Response to Lethal Encephalitis

**Figure 3:**
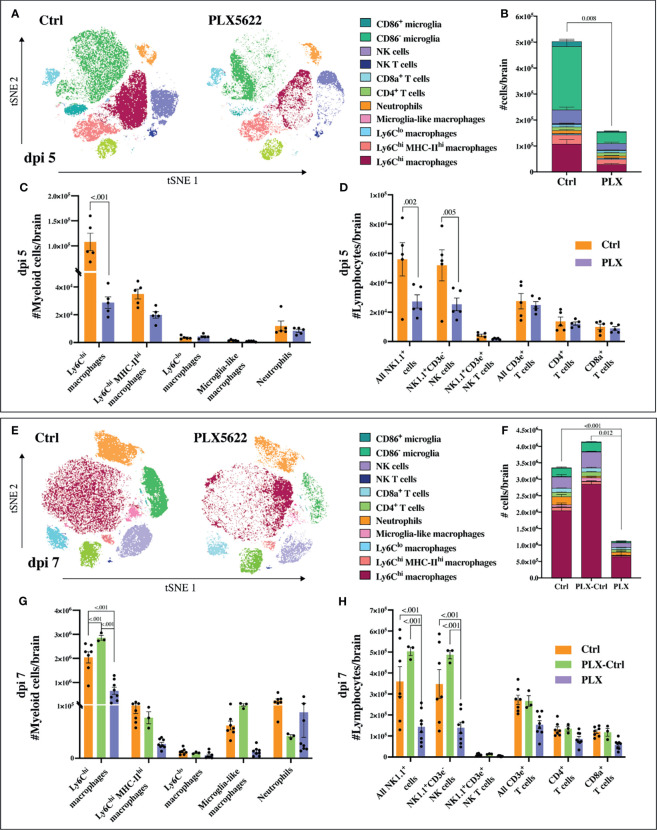
PLX5622 reduces leukocyte influx into WNV-infected brains. **(A)** tSNE plot clustered on CD45^+^ brain cells from dpi 5 *PLX* and *Ctrl* mice. **(B)** Stacked bar graph showing the number of brain cells in *PLX* and *Ctrl* mice at dpi 5. **(C, D)** Number of myeloid **(C)** or lymphoid **(D)** cells in brains of mice culled at dpi 5. **(E)** tSNE plot clustered on CD45^+^ brain cells from *PLX* and *Ctrl* mice at dpi 7. **(F)** Stacked bar graph showing the number of brain cells at dpi 7. **(G, H)** Number of myeloid **(G)** or lymphoid **(H)** cells at dpi 7 in brains of *PLX*, *PLX-Ctrl* and *Ctrl* mice. Data is presented as mean ± SEM from one (dpi 5 data) or two (dpi 7 data) independent experiments with at least three mice per group.

**Figure 4:**
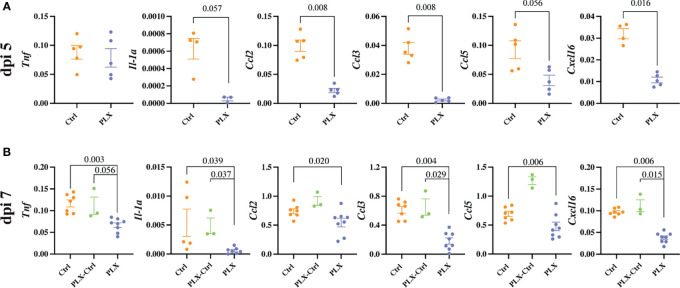
PLX5622 reduces cytokine production and the neuroinflammatory response to WNV infection. **(A, B)** Expression of select cytokines in brains of *Ctrl* and *PLX* mice at dpi 5 **(A)** and *Ctrl*, *PLX-Ctrl* and *PLX* mice at dpi 7 **(B)**, as determined by qPCR. Gene expression was normalized to the housekeeping gene, *Rpl13a*. Data is presented as mean ± SEM from one (dpi 5 data) or two (dpi 7 data) independent experiments with at least three mice per group.

Mice in the *PLX* group had reduced disease scores at dpi 7 despite higher brain viral titres (**Figures 1** and **2**), while repopulation of microglia in the *PLX-Ctrl* group abrogated this protective effect (**Figure 1**). To further investigate the ameliorative effect of PLX5622-treatment during infection, we examined single cell brain suspensions by spectral cytometry for changes in immune-cell infiltrates (**Figure 3**). In the *PLX* group at dpi 5 (**Figures 3A–D**) and 7 (**Figures 3E–H**) there was a 68% reduction in cell numbers infiltrating into infected brains compared to the *Ctrl* group. This corresponded to statistically significant decreases of 68-73% in Ly6C^hi^ macrophages and 51-60% in NK cells at dpi 5 and 7 (**Figures 3C, D, G, H**). Reduced CNS infiltration in the *PLX* group also corresponded with reduced pro-inflammatory cytokine and chemokine expression in the CNS, compared to the *Ctrl* group (**Figures 4A, B**). In contrast, in the *PLX-Ctrl* group, there was a rebound increase in the number of immune cells infiltrating into the brain and a commensurate increase in the quantity of pro-inflammatory mediators, compared to the *PLX* group (**Figures 3F–H**, **4B**). Since the number of microglia and proliferating microglia were similar to the *Ctrl* group (**Figures 1B, G, H**), the increased disease score in *PLX-Ctrl* mice (**Figure 1F**) is likely explained by the increase in CNS infiltration in this group.

Since the depletion of microglia reduced disease score, this data could suggest that microglia are pathogenic in our model. While more studies are required to confirm this, this seems unlikely considering several previously published articles investigating microglia during viral infection including WNE, have demonstrated the neuroprotective effect of microglia (31, 35–42). Moreover, we have previously shown that MCs are central to WNV immunopathology, with their reduction in the CNS improving disease phenotype and enhancing survival (6, 10). Thus, these data more likely suggest that the protective effect of PLX5622 in WNE is due to the substantial reduction in infiltrating MCs into the CNS. The reduction in NK cells is unlikely to have contributed to a decrease in disease score, as depleting these cells had no effect on this (data not shown). Reduced NK cell infiltration in *PLX* mice may be due to the reduced infiltration of MCs and reduced expression of CCL2, CCL3, CCL5 and CXCL16 in the CNS, which recruit NK cells (49). Irrespective, considering both resident and infiltrating myeloid cell populations were significantly reduced with PLX5622 treatment, it is unclear from these data which cell type principally recruits immune cells into the brain and produces these chemoattractants and pro-inflammatory mediators.

Notably, infiltrating MCs in the brains of the *PLX* group showed reduced CD11c expression and increased Ly6C expression, compared to the *Ctrl* group (**Supplementary Figure 3**). While this implied monocyte immaturity, which could be due to the absence of microglia or an off-target effect of PLX5622, as previous studies have argued (31, 38, 40), it is also possible that monocytes in the *PLX* group merely represent recently-arrived cells that have spent less time undergoing macrophage differentiation in the brain. Examining marker expression in the BM, blood and brain in the *Ctrl* group shows that on entry into the brain these cells normally upregulate CD11c and MHC-II, as well as CD11b, CX3CR1, CD64, CD68 and CD86, and downregulate Ly6C (**Supplementary Figure 4**). In mice treated with other monocyte-modulatory treatments that improve clinical outcomes and/or reduce MC infiltration into the WNV-infected CNS (6, 11), including clodronate liposomes injected intravenously at dpi 5 or anti-Ly6C antibody blockade injected intraperitoneally at dpi 5 and 6, Ly6C^hi^ macrophages also show increased Ly6C expression (**Supplementary Figure 5**), suggesting this altered phenotype is not specific to PLX5622 treatment. Thus, this MC phenotype could also represent an immature monocyte phenotype arising from the BM as it attempts to increase the output of cells during infection.

### 3.4 PLX5622 Inhibits Monocyte Production in the Bone Marrow, Thereby Reducing Their Lethal Infiltration Into the CNS

**Figure 5:**
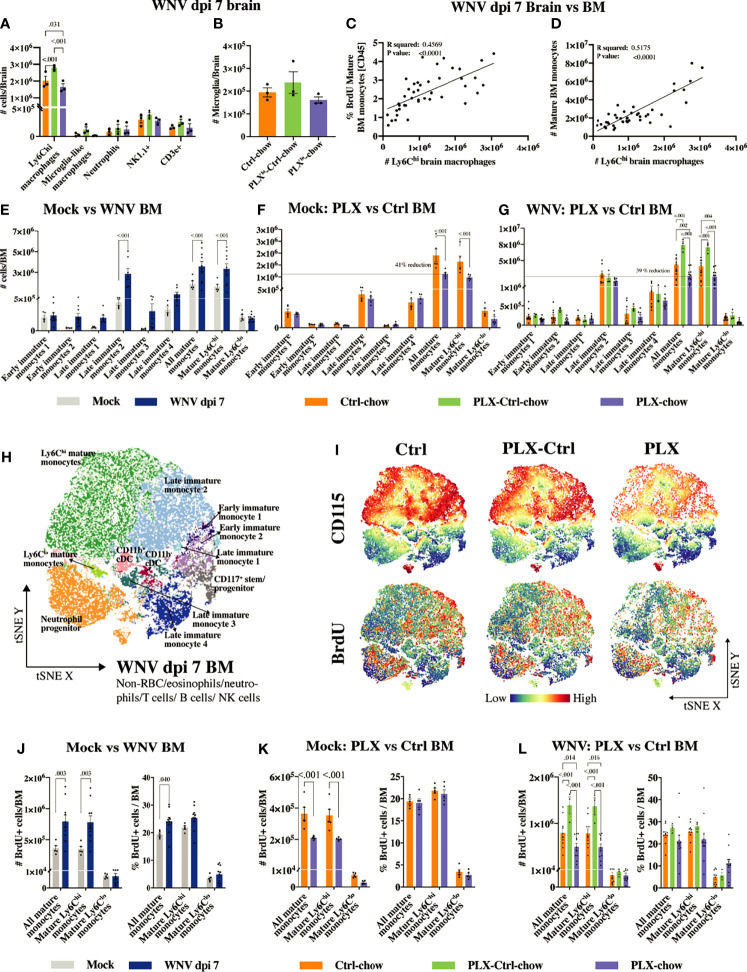
PLX5622 inhibits BM monocyte production. **(A, B)** Number of infiltrating brain cells **(A)** and microglia **(B)** at dpi 7 in mouse groups fed low-dose PLX5622: *Ctrl*, *PLX^lo^-Ctrl* and *PLX^lo^*. **(C)** Correlation between the frequency of BrdU^+^ mature BM monocytes out of CD45^+^ BM cells and the number of CNS infiltrating Ly6C^hi^ macrophages at dpi 7. **(D)** Correlation between the number of mature BM monocytes and the number of CNS infiltrating Ly6C^hi^ macrophages at dpi 7. **(E–G)** Number of myeloid cells in the BM of mock- and WNV-infected mice **(E)**, mock-infected *PLX* and *Ctrl* mice **(F)** and infected *PLX*, *PLX-Ctrl* and *Ctrl* mice **(G)**. **(H)** tSNE plot clustered on BM cells from *PLX*, *PLX-Ctrl* or *Ctrl* mice at dpi 7. **(I)** tSNE plots showing the expression of BrdU and CD115 in/on BM cells from *PLX*, *PLX-Ctrl* or *Ctrl* mice at dpi 7. **(J–L)** Number and frequency of BrdU^+^ mature BM monocytes in mock and infected mice **(J)**, mock-infected *PLX* and *Ctrl* mice **(K)** and infected *PLX*, *PLX-Ctrl* and *Ctrl* mice **(L)**. Data is presented as mean ± SEM from one **(A, B, F, K)** or three **(E, G, H–J, L)** independent experiment with at least three mice per group.

To determine whether the protective effect of PLX5622 was due to depletion of microglia or an off-target effect, we reduced PLX5622 to a dose which was insufficient to reduce microglia numbers (50, 51) by combining approximately one-sixth of the depleting dose of PLX5622 chow with control chow. Using the same feeding regimen as shown in **Figure 1A**, low-dose PLX5622 *(PLX^lo^* group*)* did not reduce microglia numbers, but reduced the number of infiltrating Ly6C^hi^ macrophages in the brain at dpi 7 by 20%, compared to the *Ctrl* group (**Figures 5A, B**). Similar to high-dose PLX5622 chow, removal of low-dose chow prior to infection at dpi 0 in the *PLX^lo^-Ctrl* group resulted in a substantial rebound effect, with a statistically significant increase in Ly6C^hi^ macrophage numbers infiltrating into the CNS, compared to the *Ctrl* group (**Figure 5A**). The low-dose chow, however, did not alter disease scores. This is likely due to the substantial number of MCs still infiltrating the CNS. Thus, since 1) microglial cell numbers in the *PLX^lo^* group remained at *Ctrl* group levels, 2) there was a reduction in CNS infiltration in the *PLX^lo^* group and 3) there was a rebound CNS Ly6C^hi^ macrophage infiltration in the *PLX^lo^-Ctrl* group (**Figure 5B**), the reduction in infiltrating cells in the brain associated with high-dose PLX5622 shown in **Figure 1F** and the corresponding decrease in disease score cannot simply be explained by the absence of microglia.

The number of Ly6C^hi^ macrophages in WNV-infected brains in the *Ctrl* group at dpi 7 correlates with both the number and proliferative status of mature Ly6C^hi^ monocytes (*i.e.* CD45^+^, Lin^-^, CD11c^lo^, CD117^-^, Sca1^int/hi^, CD48^+^, CD11b^+^, CD115^+^, Ly6C^hi^ cells) in the BM (**Figures 5C, D**). Therefore, we examined BM from mice fed the full dose of PLX5622 chow, showing the same level of microglial depletion depicted in **Figure 1**, to determine whether this produced detectable off-target effects during microglial depletion (**Figures 5E–L**). The gating strategy and phenotypic markers used to identify the various developmental stages of the myeloid lineage in the BM are shown in **Supplementary Figure 6** and **Supplementary Table 4**. Monopoiesis in the BM is substantially increased with WNV-infection, presumably to enhance the supply of “emergency” monocytes to the CNS (**Figures 5E, J**). Strikingly, CSF-1R inhibition by PLX5622 reduced the number of mature Ly6C^hi^ monocytes in the BM of both mock-infected and infected mice by ~40% (**Figures 5F, G** and **Supplementary Figures 7, 8**). This reduction in BM monocytes was also accompanied by a reduction in CSF-1R/CD115 expression (**Figure 5I**, dpi 7 BM and **Supplementary Figure 9**, mock-infected BM), as well as a reduction in the number (**Figures 5I, K, L**) and frequency (out of live cells) (**Supplementary Figures 10, 11**) of proliferating mature monocytes in the BM of PLX5622-treated mice. Interestingly, expression of CSF-1R/CD115 on mature Ly6C^hi^ monocytes correlated with number and proliferative status of these cells in the BM of *PLX* mice (**Supplementary Figure 9**), potentially providing a measure of PLX5622 efficacy in individual mice. Replacing PLX5622 with Ctrl-chow at dpi 0 in *PLX-Ctrl* mice reversed this effect, with increased CD115 expression on significantly increased numbers of BrdU^+^ mature BM monocytes at dpi 7 (**Figures 5G, I, L**). This rebound in BM monopoiesis on removal of PLX5622 likely explains the increased number of infiltrating monocytes in the brain of *PLX-Ctrl* mice. This also shows that exposure to PLX5622 does not permanently diminish the central recruitment of monocytes from the periphery, and that the migratory response of rebounding Ly6C^hi^ BM monocytes is not substantially altered.

Interestingly, despite the reduction in numbers of mature BM monocytes caused by PLX5622 in mock-infected and infected mice, the frequency of proliferating mature cells did not decrease, compared to the *Ctrl* group (**Figures 5K, L** and **Supplementary Figures 10, 11**). This suggests that a proportion of mature Ly6C^hi^ monocytes in the *PLX* group are deleted and/or not replaced. It indicates that a quorum of cells in these mice, innately resistant to PLX5622, or that become resistant to inhibition, retains the capacity to proliferate and differentiate into mature Ly6C^hi^ monocytes.

The reduction in these mature monocytes in mock-infected mice may also be explained by the reduced proliferative status of precursor cells. Indeed, in PLX5622-treated mock-infected mice, we observed a reduction in the frequency of proliferating monocyte lineage cells that we identified as early immature monocyte 1 (CD45^+^, Lin^-^, CD11c^lo^, CD117^+^, Sca1^-^, CD48^+^, CD11b^-^, CD115^hi^, Ly6C^hi^ cells) and late immature monocyte 2 (CD45^+^, Lin^-^, CD11c^lo^, CD117^-^, Sca1^-^, CD48^+^, CD11b^-^, CD115^+^, Ly6C^+^ cells) (**Supplementary Figure 10**). These are the presumptive precursors of mature Ly6C^hi^ monocytes in the BM. However, the proliferative status of these monocyte precursors in the infected BM at dpi 7 was not significantly reduced (**Supplementary Figure 11**), suggesting that infection can to some extent overcome the PLX5622-induced reduction in proliferation status seen in mock-infected animals. Thus, the data suggest that the reduction in mature Ly6C^hi^ monocytes in infected, PLX5622-treated mice is due to their deletion, while in mock-infected mice, the reduction of mature monocytes may be due to a combination of deletion and/or direct inhibition of their renewal from precursor cells.

Importantly, the number and proliferative status of classical dendritic cells (cDCs) in the BM were also reduced in mock-infected, PLX5622-treated mice (**Supplementary Figures 7, 10**). This is not surprising, since these cells also express CSF-1R (43). However, notably, these cells were unaffected by PLX5622 during infection. There was also some reduction in plasmacytoid DC numbers (pDCs) in the BM in infected PLX5622-treated mice, but this did not translate into reduced proliferation. Strikingly, however, in stark contrast to the rebound increase in monocyte proliferation and numbers seen in the BM of the *PLX-Ctrl* group, pDCs underwent a significant reduction in proliferation after removal of PLX5622 in this group, a response that was also observed in MHC-II^lo^ B cells. Other cell types in the spleen and BM were not reduced with PLX5622 treatment (**Supplementary Figures 7, 12**). Taken together, PLX5622 has non-microglial effects on proliferation and renewal of various immune subsets. Importantly, the effect on the myeloid lineage evidently results in the reduction of monocyte immigration into the CNS, ameliorating the severe inflammation associated with infection.

### 3.5 Antibody Blockade of CSF-1R, Like PLX5622, Reduces the Inflammatory Recruitment of MCs Into the WNV-Infected Brain by Impacting BM Monopoiesis

**Figure 6:**
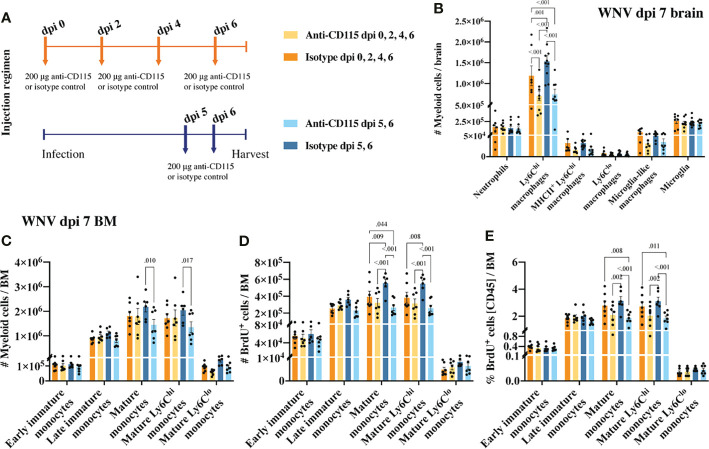
Treatment with anti-CSF-1R, like PLX5622, reduces CNS infiltration and BM monocyte production. **(A)** Schematic of experimental workflow. Mice were infected with WNV and treated with an isotype control monoclonal blocking antibody or anti-CSF-1R at either 1) dpi 0, 2, 4 and 6 or 2) dpi 5 and 6. **(B)** Number of myeloid cells in brains of mice treated with an isotype control or anti-CSF-1R at WNV dpi 7. **(C)** Number of myeloid cells in infected (dpi 7) BMs from mice treated with an isotype control or anti-CSF-1R. **(D, E)** Number **(D)** and percent **(E)** of BrdU^+^ myeloid cells in infected (dpi 7) BMs from mice treated with an isotype control or anti-CSF-1R. Data is presented as mean ± SEM from two independent experiment with at least six mice per group.

Given PLX5622 targets CSF-1R, we used monoclonal anti-CSF-1R antibody blockade to determine if this approach could be used therapeutically. Since monoclonal antibodies (mAb) do not cross the intact BBB, this would protect microglia from adventitious effects of CSF-1R inhibition. Treatment of mice with anti-CSF-1R mAb at dpi 0, 4, 5 and 6 or at dpi 5 and 6 resulted in a striking ~50% reduction in the number of Ly6C^hi^ macrophages infiltrating the WNV-infected CNS without affecting numbers of microglia (**Figures 6A, B**). However, unlike PLX5622, the anti-CSF-1R mAb did not cause a reduction in disease score, nor increase survival in experiments using a 50% lethal dose of WNV (data not shown). Thus PLX5622 may be a more potent inhibitor of CSF-1R than the anti-CSF-1R mAb and, as a small molecule, better able to inhibit autocrine actions of endogenous CSF-1, which may not be amenable to mAb blockade (52).

Treating infected mice with anti-CSF-1R antibody not only significantly reduced CNS infiltration, but also impacted BM monopoiesis similar to PLX5622 (**Figures 6C–E**). Interestingly, treating mice only twice in the later phase of disease had a greater impact on BM monocytes than treating mice every second day from dpi 0 (**Figures 6C–E**). Perhaps, alternate mechanisms came into play to compensate for CSF-1R inhibition in mice treated for an extended period with anti-CSF-1R. Nevertheless, considering anti-CSF-1R had a similar effect on CNS infiltration and BM monocyte production, this suggests that the effect we saw with PLX5522 is likely due to inhibition of CSF-1R and not other tyrosine kinase receptors.

**Figure 7:**
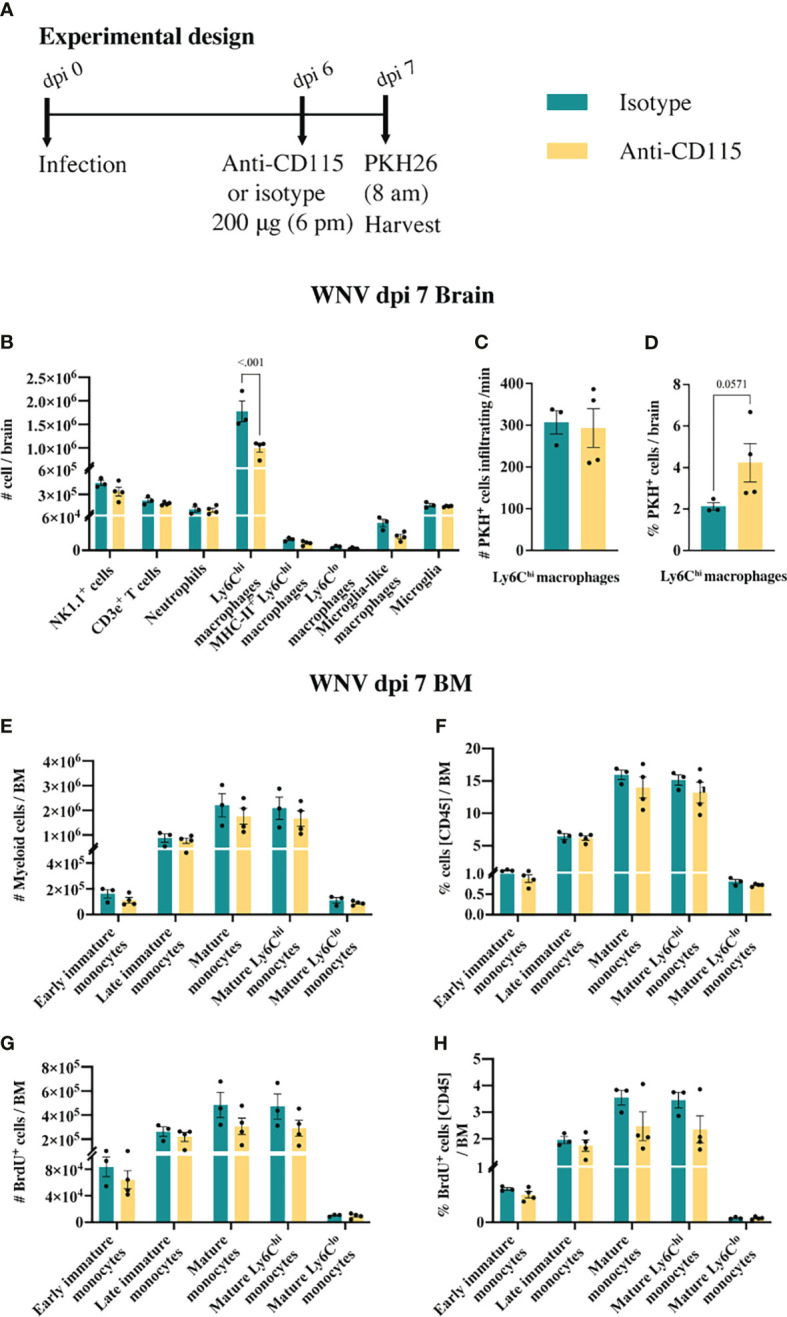
Anti-CSF-1R does not affect monocyte trafficking into the CNS. **(A)** Schematic of experimental workflow. Mice were infected and treated with an isotype control monoclonal blocking antibody or anti-CSF-1R. Two hours prior to tissue collection animals were injected with an intravenous dye. **(B)** Number of myeloid and lymphoid populations in brains of mice infected with WNV and treated with an isotype control antibody or anti-CSF-1R. **(C)** Number of PKH26^+^ Ly6C^hi^ macrophages infiltrating the WNV-infected brain per minute at dpi 7. **(D)** Percent of PKH26^+^ Ly6C^hi^ macrophages in WNV-infected brains at dpi 7. **(E, F)** Number **(E)** and percent **(F)** of myeloid cells in infected (dpi 7) BMs from mice treated with an isotype control or anti-CSF-1R. **(G, H)** Number **(G)** and percent **(H)** of BrdU^+^ myeloid cells in infected (dpi 7) BMs from mice treated with an isotype control or anti-CSF-1R. Data is presented as mean ± SEM from one independent experiment with at least three mice per group.

CSF-1R mAb blockade effectively reduced MC infiltration into the CNS, even when administered in the later phase of disease (**Figure 6B**). This suggests that CSF-1R blockade can rapidly inhibit BM monocyte production. However, considering previous studies have suggested a role for CSF-1 in monocyte migration (52–54), this data does not exclude the possibility that anti-CSF-1R modulates monocyte migration into the brain. To investigate this, infected mice, treated with anti-CSF-1R 14 hours previously, were injected intravenously with the membrane dye, PKH26, for 2 h prior to tissue collection (**Figure 7A**). In 16 hours, anti-CSF-1R had significantly reduced MC numbers in the brain (**Figure 7B**). However, the numbers of dye-positive Ly6C^hi^ macrophages recently infiltrating the brains of anti-CSF-1R-treated mice were similar to those seen in untreated mice (**Figure 7C**), suggesting that anti-CSF-1R blockade does not impact monocyte trafficking into the brain. Interestingly, the proportion of dye-positive Ly6C^hi^ macrophages was somewhat higher in anti-CSF-1R-treated mice (**Figure 7D**), presumably because anti-CSF-1R mAb-treated animals had a lower overall infiltration of MCs into the brain than the isotype control mAb-treated mice. Notably, we observed no change in the number or proportion of any BM monocyte subsets (**Figures 7E, F**). In this short period, however, the proportions of mature Ly6C^hi^ monocytes incorporating BrdU were reduced, albeit this was not statistically significant (**Figures 7G, H**). This indicates that CSF-1R blockade does not inhibit immigration of these cells into the brain and suggests that the reduced CNS recruitment of MCs in WNV-infected mice during CSF-1R inhibition is due to reduced monopoiesis and/or deletion.

## 4 Discussion

The precise temporal functions of microglia in the acute phase of viral infection are ill-defined. Historically, the limited tools to identify microglia accurately in the inflamed brain have made it challenging to study microglia-specific responses to CNS perturbation. The development of the microglia-depleting agent, PLX5622, has facilitated exploration of microglial functions in disease. However, many studies are predicated on this agent being microglia-specific, while few have examined its possible indirect or off-target effects. Here we show that as well as depleting both microglia and a sub-population of BAMs in the CNS, PLX5622 also inhibited mature BM monocyte proliferation, reducing their lethal recruitment into the virus-infected CNS, thereby reducing severe neuroinflammation in WNV infection and the associated disease phenotype. Monoclonal antibody blockade targeting CSF-1R late in disease also reduced BM monocyte production and infiltration of these cells into the inflamed CNS, suggesting that the effect of PLX5622 is *via* inhibition of CSF-1R and not other kinase targets. Antibody blockade however, did not alter disease scores, suggesting higher potency and therapeutic potential of PLX5622 over anti-CSF-1R in monocyte-mediated diseases.

We showed that PLX5622 inhibits the production of mature BM monocytes, reducing the number of “emergency” cells that can be recruited to the inflamed brain (55). Previous studies failed to demonstrate any impact on peripheral myeloid cells even after 7 months of treatment with PLX5622 (33), although these studies were focused on circulating blood leukocytes and the phenotypic markers used to examine the BM compartment were insufficient to identify the various BM populations detailed here (31, 33, 35, 38). While some studies have reported effects on peripheral populations in the spleen and blood, this is inconsistent across publications. One study demonstrated a reduction in CSF-1R^+^ monocytes in the blood of PLX5622-treated mice (37), but this was not observed in others (33, 35, 38). Furthermore, none of these studies investigated cell proliferation to determine the potential impact of PLX5622 on monopoiesis. Recently, this agent was shown to suppress the proliferative capacity of CX3CR1^+^ BM and splenic ‘macrophages’ (44). However, cells were isolated from mice 3 weeks after PLX5622 cessation and stimulated *ex vivo* for 5 days, which is unlikely to reflect the acute *in vivo* context.

We demonstrated the impact of PLX5622-mediated CSF-1R inhibition on the proliferation of Ly6C^hi^ mature monocytes *in vivo* in the BM of infected and mock-infected mice and in cDCs in the BM of mock-infected animals. Early work showed that BM-derived macrophages require CSF-1 throughout G1 to enter S phase (4), while at low CSF-1 concentrations they enter G0/G1 to become quiescent. Our data are consistent with the expression of CSF-1R by progenitors, monocytes, macrophages, and cDCs (43), and the reliance of monocytes on CSF-1R signaling for survival, differentiation and proliferation (2–5, 56). Interestingly, there was no reduction in numbers of Ly6C^lo^ mature BM monocytes in PLX5622-treated BM, presumably because these cells can be derived independently of Ly6C^hi^ monocytes in the BM, unlike in the blood (7). Importantly, also, PLX5622 had no impact on numbers and proliferative status of neutrophils and lymphocytes in the BM, nor on monocytes or macrophages in the spleen, which are evidently maintained independently of CSF-1R signaling. Alternatively, in the CNS, the same proportion of microglia remaining in *PLX*-mice continued to proliferate at dpi 5. This suggests that microglia populations may be differentially dependent on CSF-1R signaling or perhaps an alternate CSF-1R ligand, IL-34 can act on a different, currently undefined receptor (52).

In our model, PLX5622 was 20% more effective than the anti-CSF-1R mAb at reducing the number of infiltrating Ly6C^hi^ monocytes into the WNV-infected brain. This may explain why PLX5622 reduced disease scores, while the anti-CSF-1R mAb did not. The effectiveness of PLX5622 may rely on its ability to block autocrine actions of endogenous CSF-1, further inhibiting monocyte production for recruitment into the CNS (52, 57), whereas endogenous CSF-1 is inaccessible to mAb. While future investigations are required to confirm this, the depletion of microglia which occurs in PLX5622-treated mice and not mAb-treated mice is unlikely to explain the protective effect of PLX5622, as multiple studies have shown that microglia are neuroprotective during viral infection (31, 36–40). The enhanced effectiveness of PLX5622 also cannot be explained by the extended period of time in which mice were treated with PLX5622, compared to anti-CSF-1R mAb. Mice treated for two days with anti-CSF-1R reduced monocyte production in the BM and MC immigration into the CNS just as effectively as mice treated for a week with anti-CSF-1R. It was suggested that non-specific targeting of KIT and FLT3 by PLX5622 may explain the reduced proliferation of ‘macrophages’ from PLX5622-treated mice (58). However, in our experiments, anti-CSF-1R mAb and PLX5622 had a similar effect on the BM, suggesting that the inhibitory effect of PLX5622 is likely *via* the CSF-1R.

In apparent contradiction to our report, anti-CSF-1R blockade was shown to specifically deplete Ly6C^lo^ monocytes in the blood without affecting populations in the BM, although this study used a different experimental approach (59). We did not examine the peripheral blood in our study, however, our use of high-dimensional cytometry enabled accurate identification of these BM populations. Since Ly6C^lo^ monocytes are strictly derived from Ly6C^hi^ monocytes in the blood (7), this discrepancy could simply be explained by the reduction of Ly6C^hi^ monocytes in the BM, as shown in this report, which would in turn lead to the observed reduction in Ly6C^lo^ monocytes in the blood.

Removal of PLX5622 prior to infection resulted in a rebound effect where BM monocyte production and CNS infiltration was enhanced. This may be because Ly6C^hi^ monocytes normally act as a “sink” for CSF-1 (7), clearing it *via* receptor-mediated endocytosis upon binding to CSF-1R (5, 60). The loss of this sink either by depletion of these cells or by CSF-1R blockade could thus result in increased CSF-1 levels (7, 59). Following removal of PLX5622, high CSF-1 levels could prompt rebound monopoiesis *via* enhanced CSF-1R signalling, which would enable cells to re-enter the growth cycle (4). Downregulation of CSF-1R on BM monocytes and DCs in the BM of PLX5622-treated mice is consistent with this, as saturation of the CSF-1R induces internalization and degradation of the receptor-ligand complex resulting in “downmodulation” (61). This rapid rebound monopoiesis, demonstrates that PLX5622 reversibly modulates BM monocytes, which may make this drug a viable therapeutic treatment in diseases in which monocytes play a detrimental role, especially if used at a lower dose at which microglia are minimally depleted (50, 51) or the drug were engineered to reduce its capacity to cross the BBB to protect microglia.

In contrast to other studies investigating viral infection (31, 36–40), PLX5622-mediated microglia depletion in WNV infection in our model, ameliorated the disease phenotype. Other groups have demonstrated enhanced mortality associated with PLX5622-mediated microglia depletion during WNV infection, as well as a range of other viral infections. This discrepancy is likely due to the different infection models used, with other groups using alternative virus strains, doses and inoculation routes which are non-lethal and/or less inflammatory. The disparate genders, ages and strains of mice that have been used by other groups may also play a role. For instance, Seitz et al. (36) inoculated Swiss-Webster 7–10-week-old female mice with a pathogenic strain of WNV (WNV-NY99) in the periphery *via* a footpad inoculation, while Funk and Klein (38) used three models but primarily investigated C57BL/7 6-week-old males intracranially inoculated with the attenuated virus, WNV-NS5- E218A. In contrast, we inoculated the Sarafend stain of WNV (a lineage II strain of WNV) intranasally, which directly infects the CNS *via* the olfactory nerve (6). In this model, blocking the infiltration of Ly6C^hi^ BM-derived monocytes into the CNS by various means reduces disease signs and increases survival, demonstrating the pathogenic contribution of these cells to disease progression (6, 8, 10, 11). Although a specific microglia depletion method is required to confirm the role of microglia in our model of WNE, according to previous findings, including 1) the contribution of MCs to mortality in WNE (6, 8, 10, 11) and 2) the neuroprotective role of microglia during viral infection (31, 35–42), microglia are more likely to play protective role, as previously demonstrated. Since the infiltration of MCs into the CNS is more pathogenic in our model than the absence of the supposed protective microglial functions in WNV-infection, we did not observe an accelerated mortality or disease score. Thus, the reduced disease score in PLX5622-treated infected mice is likely due to the substantial reduction in CNS macrophages. Similarly, while the increased disease score seen in *PLX-Ctrl* mice is likely due to enhanced CNS infiltration of these cells, repopulating microglia may however, also exacerbate the disease phenotype by playing a pathogenic role, although this remains to be investigated.

Microglia may play a protective role in our model as previously suggested (31, 36–40). Indeed, similar to earlier reports (31, 35, 36, 38), microglia depletion in our model increases viral load in the CNS, while increasing numbers of microglia correlate with decreased viral RNA, suggesting a role for these cells in viral clearance. Although other cells may also contribute to viral control in our model, since we only observed a modest increase in viral load with microglia depletion. Interestingly, both the remaining microglia in *PLX* mice and the repopulating microglia in *PLX-Ctrl* mice expressed lower levels of the nominal microglia-specific marker, P2RY12. As P2RY12 is required for phagocytosis of virus-infected neurons in PRV infection (35), this suggests that non-ablated microglia in *PLX*-mice and newly repopulated microglia are poorly-equipped to control pathogen invasion and might explain the increased viral loads observed in the brains of these mice.

Similar to other studies investigating viral encephalitis, we showed that Ly6C^hi^ monocytes infiltrating the WNV-infected brain adopt an altered phenotype in mice fed PLX5622, specifically showing higher Ly6C expression. Other studies have shown a reduced expression of CD86 (38) or MHC-II (31, 40), as well as increased Ly6C expression (31, 41) on infiltrating macrophages in WNV, JHMV, MHV and TMEV infection. This “immature macrophage” phenotype was thought to be due to a combination of the absence of microglia and potential indirect effects caused by PLX5622. We showed that two other monocyte modulatory treatments in WNE resulted in an MC phenotype similar to those seen in PLX5622-treated brains. This rules out the absence of microglia and/or PLX5622 treatment as the specific cause. It seems more likely that increased expression of Ly6C may represent a shift to emigration of a less mature monocyte as the BM attempts to increase the output of these cells in “emergency” conditions. Increased Ly6C expression has previously been suggested to be a marker of MC immaturity (62–64), although in IRF8 KO mice in which monocyte production is inhibited, the predominance of Ly6C^int^ cells suggests a more immature monocyte phenotype (65). The function of Ly6C remains somewhat elusive, although the ability of anti-Ly6C treatment to reduce MC recruitment into the CNS, demonstrates a clear role for this marker in immigration (11). The increased expression of this marker on MCs in the brain may thus also denote cells that have most recently traversed the endothelium into the CNS parenchyma, as this marker is downregulated with time spent in the brain (6, 12), skin (66) and lung (67).

In summary, off-target effects produced by PLX5622 make it impossible to completely isolate the role of microglia in CNS infection. We show that PLX5622 affects a subpopulation of BAMs in the brain and monocytes and DCs in the BM. Serendipitously, however, the inhibition of mature BM monocyte proliferation reduced the recruitment of “emergency” monocytes into CNS, substantially reducing neuroinflammation and disease score. Used at doses that minimally deplete microglia or modified to reduce passage across the BBB, this work highlights for the first time, the potential therapeutic value of PLX5622 in viral infection, as well as other monocyte-mediated diseases. Although PLX5622 provides a valuable tool in microglia research, our work suggests that significant re-evaluation will be necessary to take into account the non-microglial affects caused by this molecule.

## Data Availability Statement

The raw data supporting the conclusions of this article will be made available by the authors, without undue reservation.

## Ethics Statement

All experiments were performed in accordance with National Health and Medical Research Council’s ethical guidelines with the animal ethics approval number 2019/1696, reviewed and approved by the University of Sydney Animal Ethics Committee.

## Author Contributions

Investigation, formal analysis, formal data curation, methodology and visualisation (AS), assistance with qPCR (DN), assistance with plaque assay (ZL), conceptualization and writing – original draft (AS & NK), writing – review & editing (AS, DN, LM, IC, MH & NK) and funding acquisition (NK). All authors contributed to the article and approved the submitted version.

## Funding

NK was supported by a National Health and Medical Research Council Project Grant 1088242 and the Merridew Foundation. AS is supported by the Australian Government Research Training Stipend Scholarship and The University of Sydney Postgraduate Merit Award.

## Conflict of Interest

The authors declare that the research was conducted in the absence of any commercial or financial relationships that could be construed as a potential conflict of interest.

## Publisher’s Note

All claims expressed in this article are solely those of the authors and do not necessarily represent those of their affiliated organizations, or those of the publisher, the editors and the reviewers. Any product that may be evaluated in this article, or claim that may be made by its manufacturer, is not guaranteed or endorsed by the publisher.
