# Supplementary material for: PLX5622 Reduces Disease Severity in Lethal CNS Infection by Off-Target Inhibition of Peripheral Inflammatory Monocyte Production
Source: Front Immunol. 2022 Mar 25;13:851556. doi: 10.3389/fimmu.2022.851556 (PMC8990748; doi:10.3389/fimmu.2022.851556)
Supplement: Supplementary file 1 [file DataSheet_1.pdf]

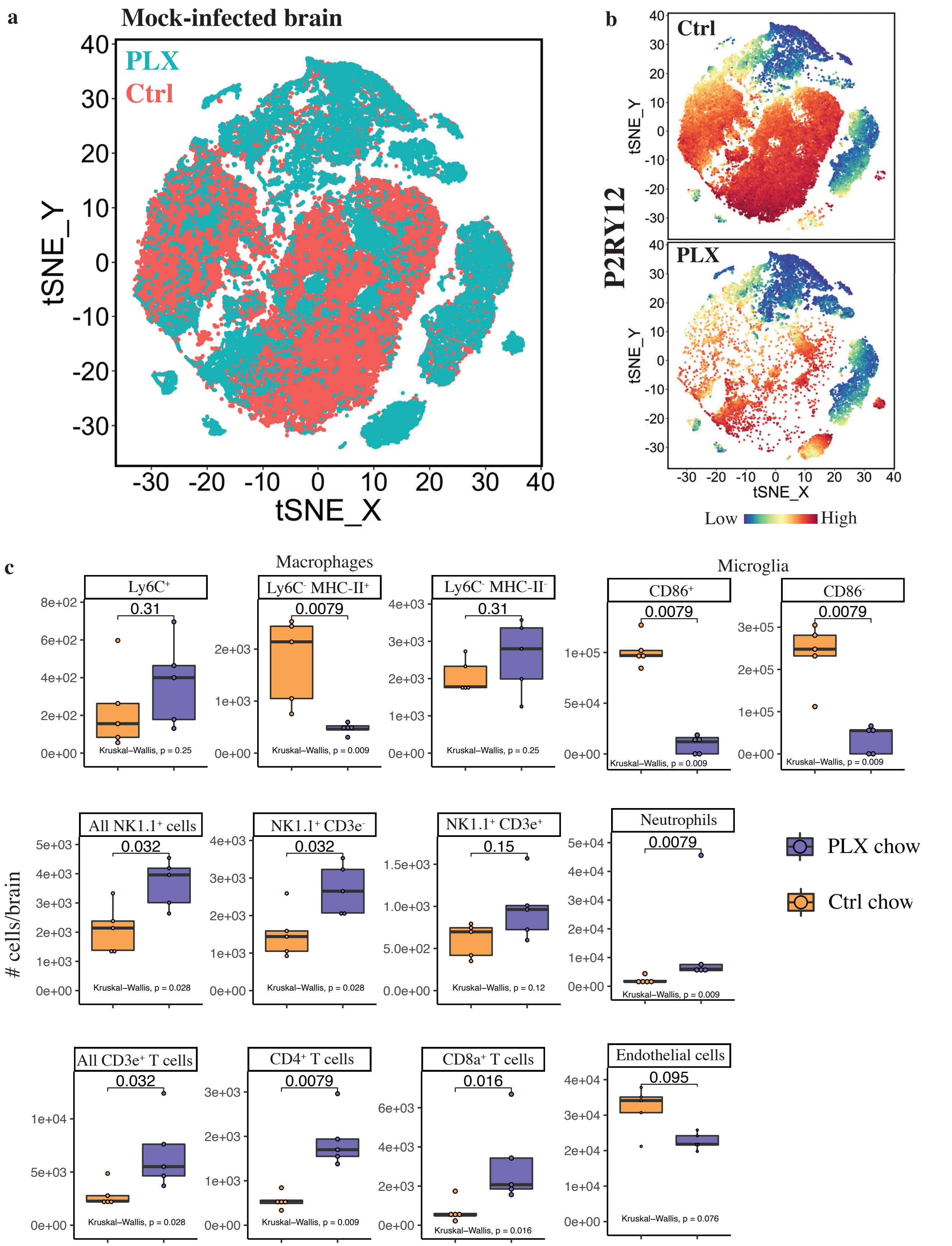

Supplementary Figure 1: PLX5622 produces non-microglial specific effects in the mock-infected brain. (a) tSNE plot clustered on brain cells from mock-infected PLX and mock-infected Ctrl mice. (b) tSNE plot showing the expression of P2RY12 on brain cells from mock-infected PLX and mock-infected Ctrl mice. (c) Box plots showing the number of brain cells in mock-infected PLX and mock-infected Ctrl mice. Data is presented as mean ± SEM from one independent experiment with five mice per group. p values were determined using a Mann-Whitney test.

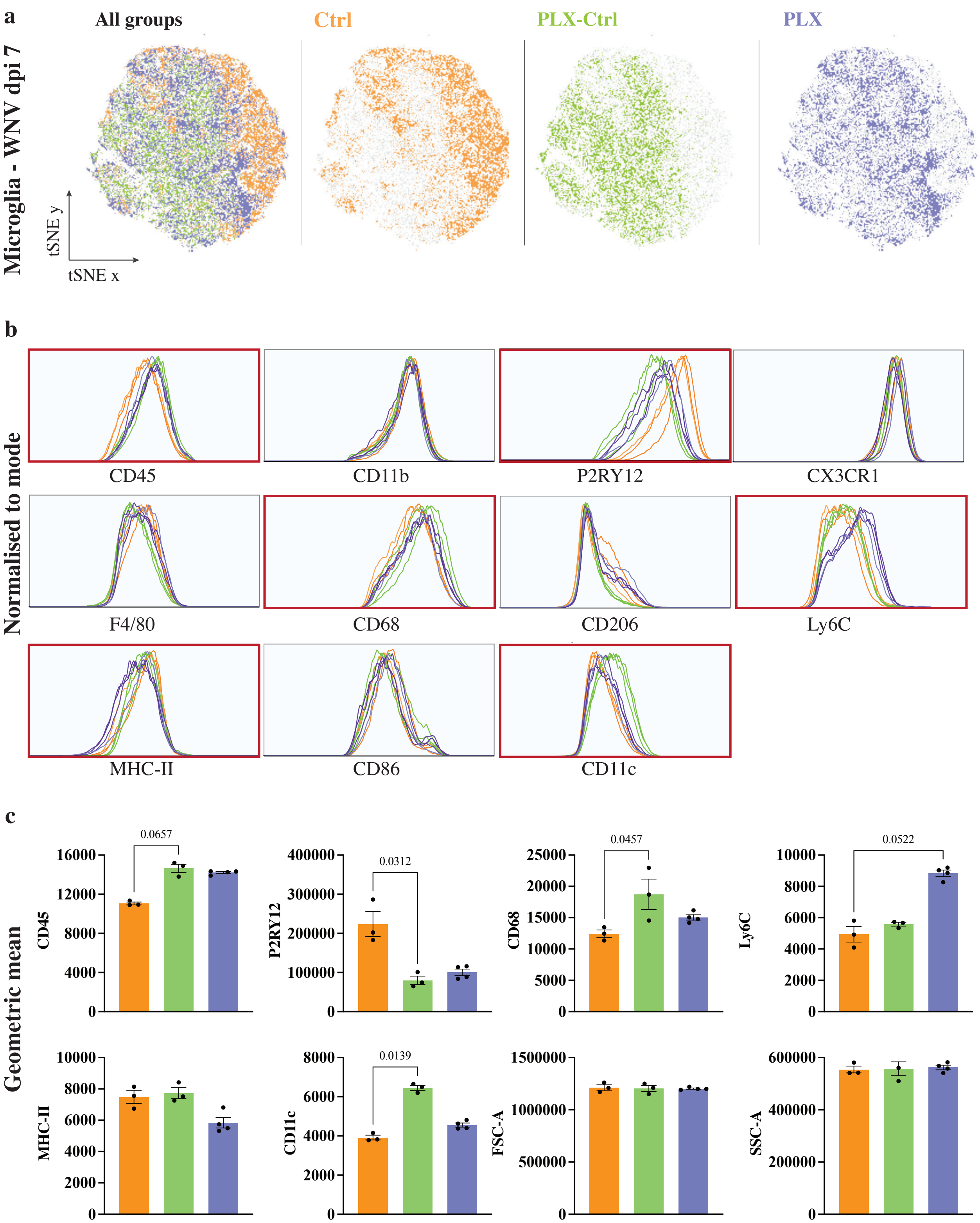

Supplementary Figure 2: Phenotypic changes in repopulated microglia and microglia remaining in PLX5622-treated mice. (a) tSNE plot clustered on microglia from Ctrl, PLX-Ctrl and PLX mice at dpi 7. (b, c) Histograms (b) and bar graphs (c) showing the geometric mean of select markers on/in microglia from dpi 7 mice. Statistical analysis was performed on markers outlined in red on histograms. Data show mean ± SEM from two independent experiments. p values were determined using a Kruskal-Wallis test and Dunn's multiple comparisons test.

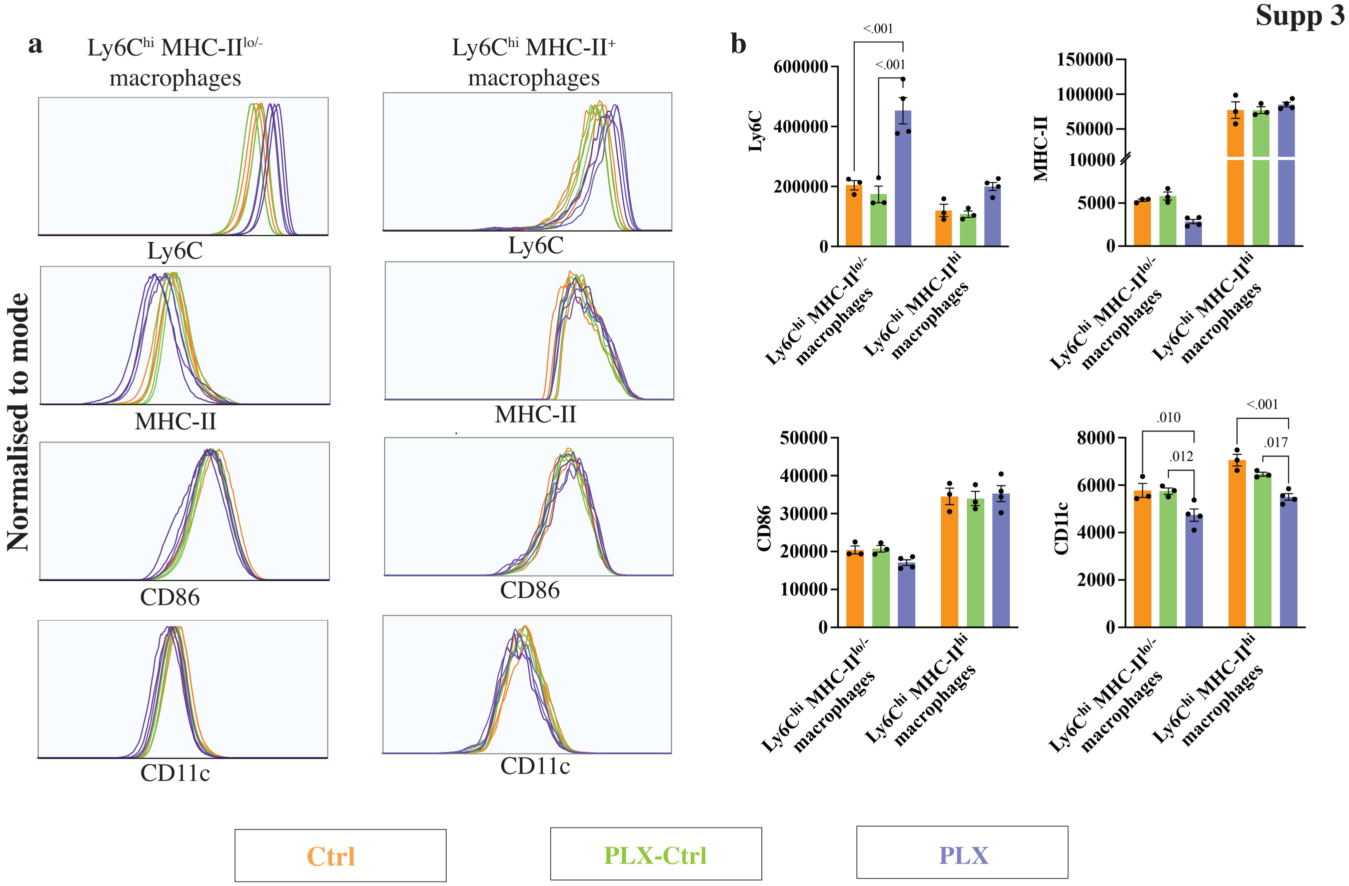

Supplementary Figure 3: Macrophages infiltrating PLX5622-treated and WNV-infected mice brains have a higher expression of Ly6C. (a, b) Histograms (a) and bar graphs (b) showing the geometric mean of select markers on infiltrating macrophages populations in the CNS of Ctrl, PLX-Ctrl and PLX mice at dpi 7. Data shows mean ± SEM and is representative of two independent experiments. p values were determined using a Kruskal-Wallis test and Dunn's multiple comparisons test.

a Ly6C^{hi} monocyte/macrophage marker expression across tissue

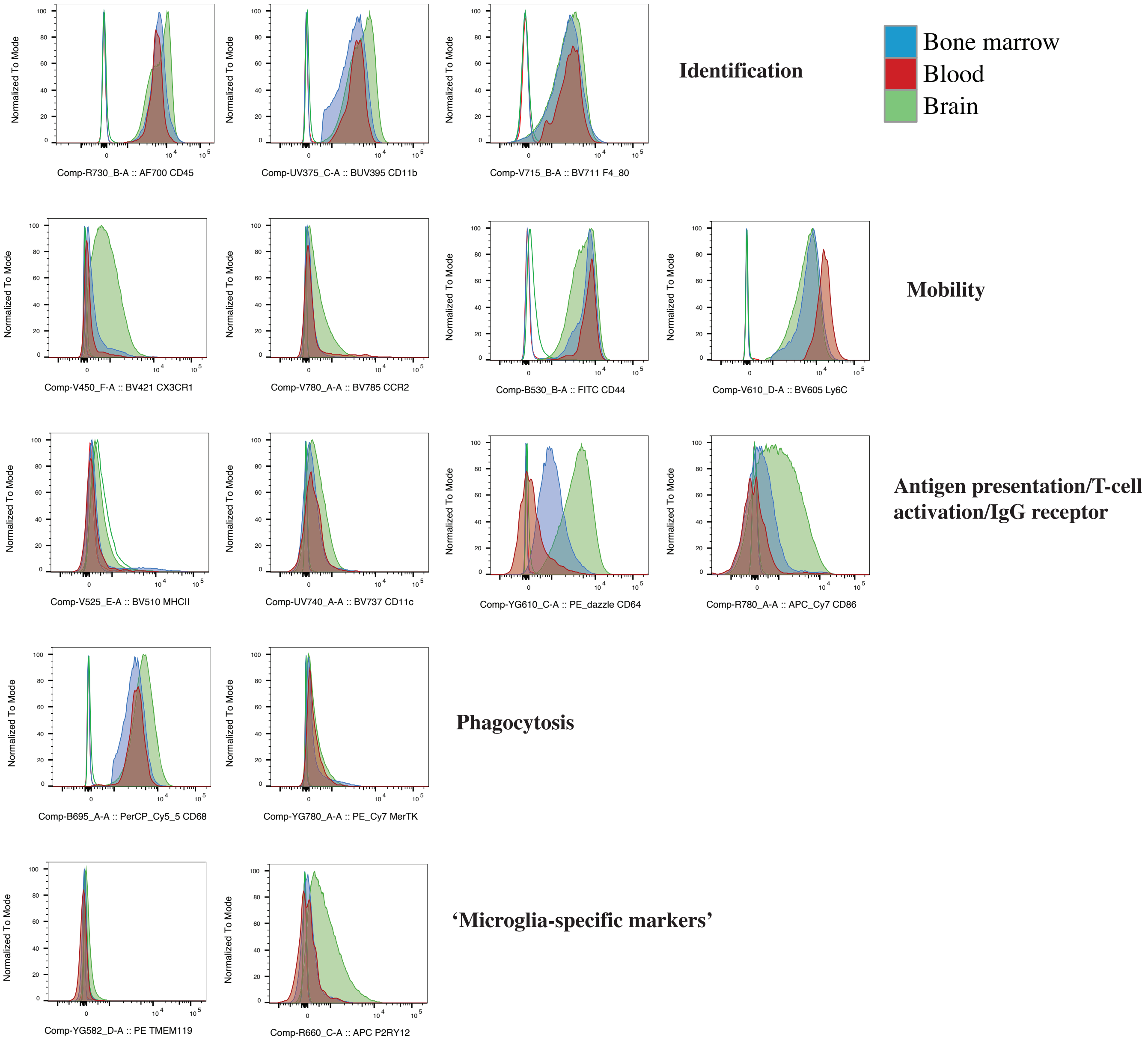

b

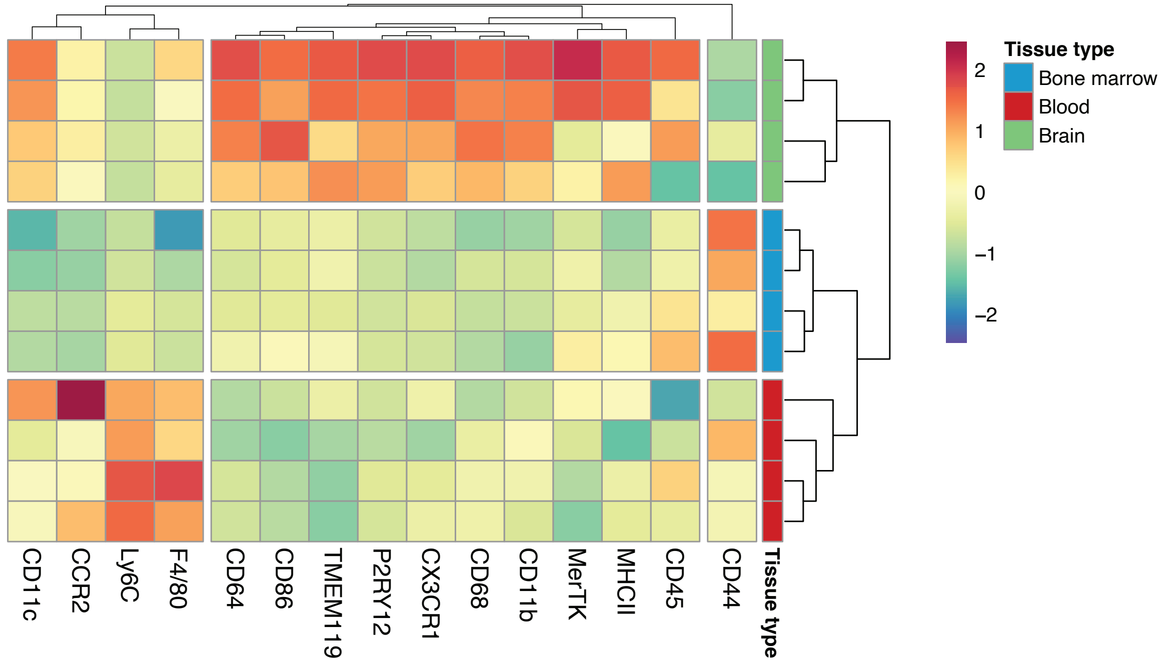

Supplementary Figure 4: Phenotypic differentiation of BM-derived macrophages in the WNV-infected CNS. (a, b) Histograms (a) and heatmap (b) showing the expression of select markers on Ly6Chi monocytes/ macrophages in the blood, BM and brain of WNV dpi 7 mice.

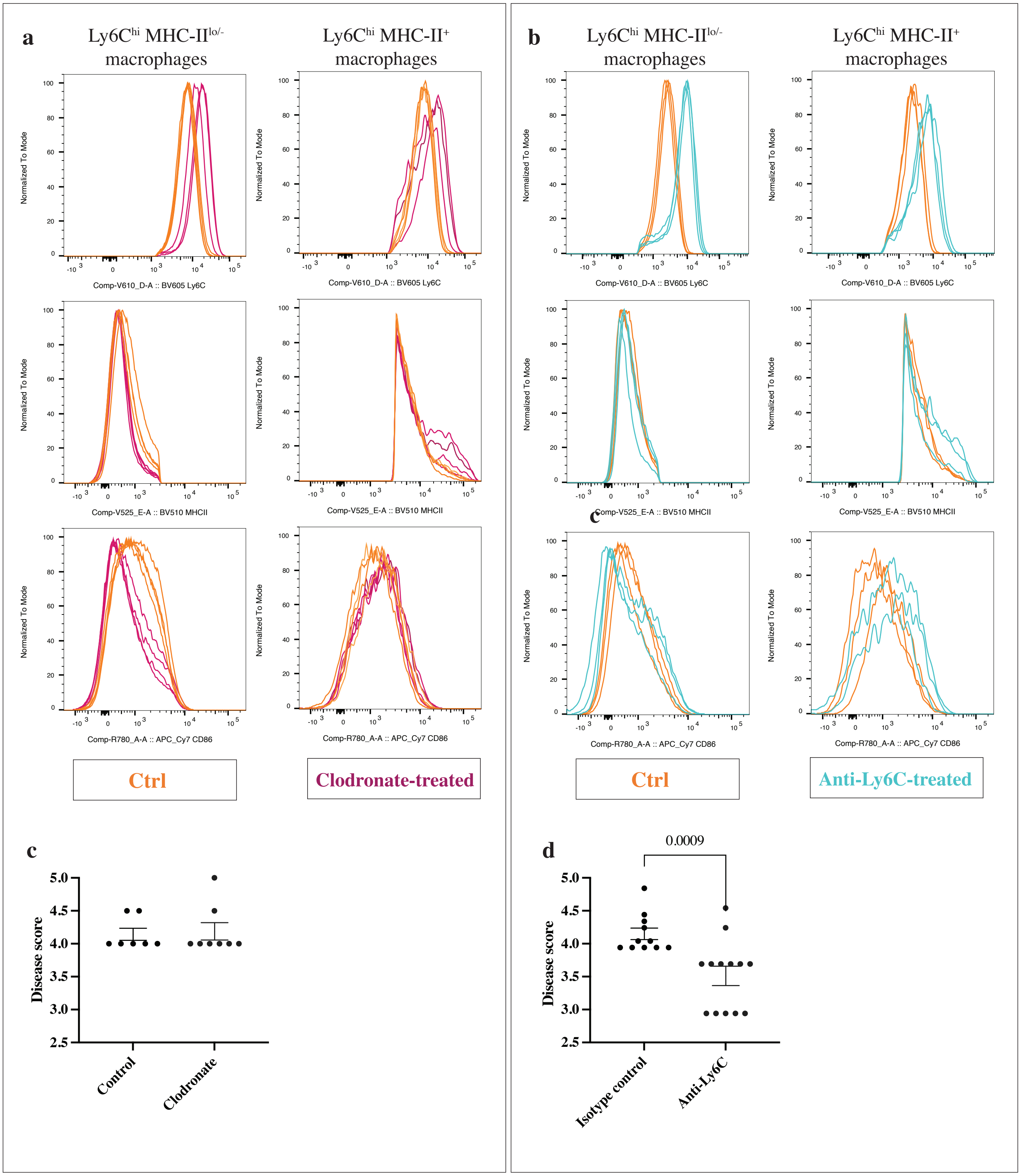

Supplementary Figure 5: Macrophages infiltrating the CNS of WNV-infected mice treated with clodronate liposomes or anti-Ly6C blocking antibody have a higher expression of Ly6C. (a, b) Histograms showing the expression of select markers on Ly6Chi macrophages in the brain of WNV-infected mice at dpi 7 treated with clodronate liposomes (a) or anti-Ly6C blocking antibody (b). (c, d) Disease score of WNV-infected mice at dpi 7 treated with clodronate liposomes (c) or anti-Ly6C blocking antibody (d).

Supp 6

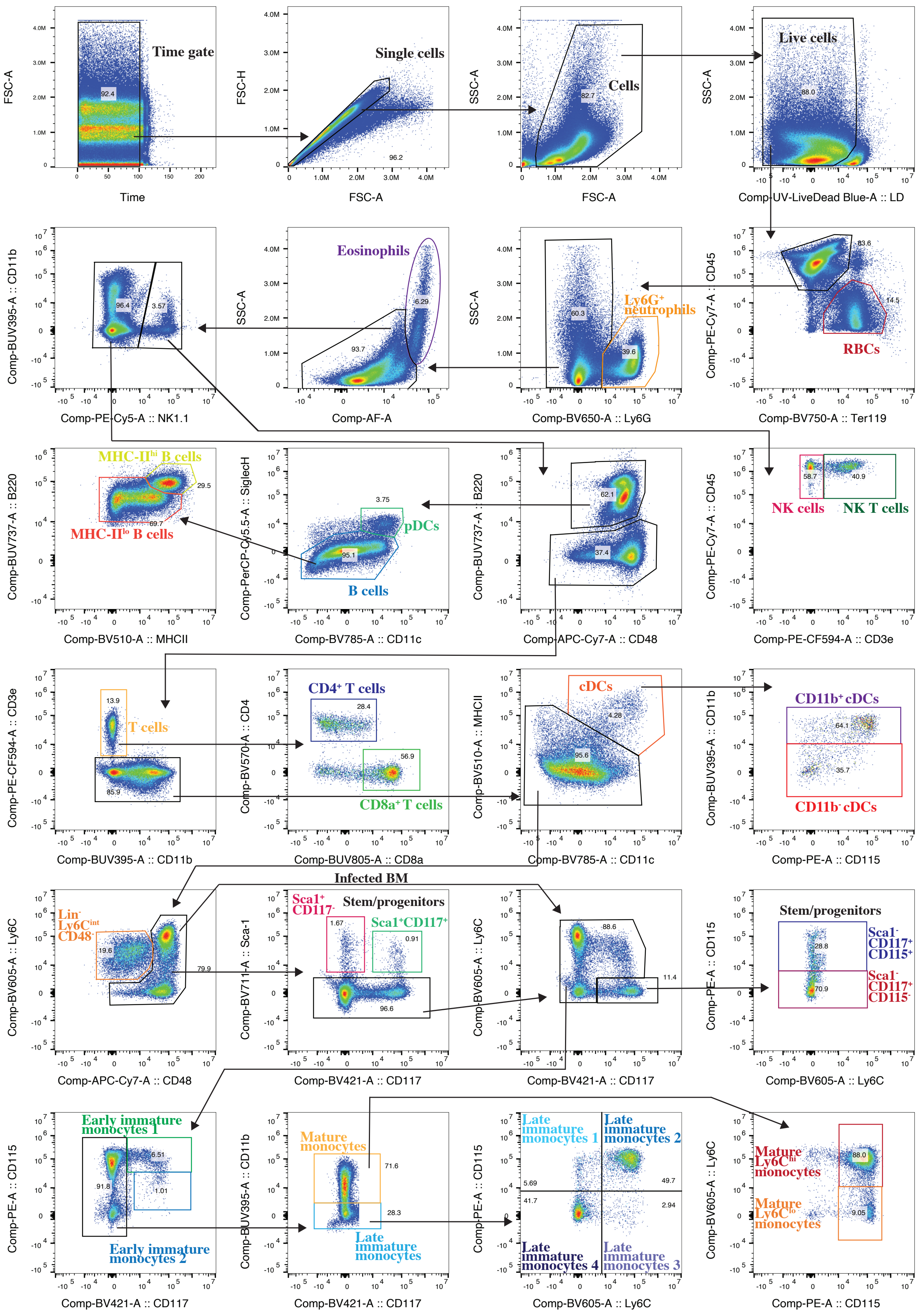
Time gate
92.4
FSC-A
Time
Single cells
96.2
FSC-H
FSC-A
Cells
82.7
SSC-A
FSC-A
Live cells
88.0
SSC-A
Comp-UV-LiveDead Blue-A :: LD
96.4
3.57
Comp-BUV395-A :: CD11b
Comp-PE-Cy5-A :: NK1.1
Eosinophils
6.29
93.7
SSC-A
Comp-AF-A
60.3
Ly6G+ neutrophils
39.6
SSC-A
Comp-BV650-A :: Ly6G
83.6
14.5
RBCs
Comp-PE-Cy7-A :: CD45
Comp-BV750-A :: Ter119
MHC-IIhi B cells
29.5
69.7
MHC-IIlo B cells
Comp-BUV737-A :: B220
Comp-BV510-A :: MHCII
3.75
pDCs
95.1
B cells
Comp-PerCP-Cy5.5-A :: SiglecH
Comp-BV785-A :: CD11c
62.1
37.4
Comp-BUV737-A :: B220
Comp-APC-Cy7-A :: CD48
58.7
40.9
NK cells
NK T cells
Comp-PE-Cy7-A :: CD45
Comp-PE-CF594-A :: CD3e
13.9
T cells
85.9
Comp-PE-CF594-A :: CD3e
Comp-BUV395-A :: CD11b
CD4+ T cells
28.4
56.9
CD8a+ T cells
Comp-BV570-A :: CD4
Comp-BUV805-A :: CD8a
cDCs
4.28
95.6
Comp-BV510-A :: MHCII
Comp-BV785-A :: CD11c
CD11b+ cDCs
64.1
35.7
CD11b- cDCs
Comp-BUV395-A :: CD11b
Comp-PE-A :: CD115
Infected BM
Lin- Ly6Cint CD48-
19.6
79.9
Comp-BV605-A :: Ly6C
Comp-APC-Cy7-A :: CD48
Sca1+ CD117-
Stem/progenitors
Sca1+CD117+
1.67
0.91
96.6
Comp-BV711-A :: Sca-1
Comp-BV421-A :: CD117
88.6
11.4
Comp-BV605-A :: Ly6C
Comp-BV421-A :: CD117
Stem/progenitors
28.8
70.9
Sca1- CD117+ CD115+
Sca1- CD117+ CD115-
Comp-PE-A :: CD115
Comp-BV605-A :: Ly6C
Early immature monocytes 1
6.51
91.8
1.01
Early immature monocytes 2
Comp-PE-A :: CD115
Comp-BV421-A :: CD117
Mature monocytes
71.6
28.3
Late immature monocytes
Comp-BUV395-A :: CD11b
Comp-BV421-A :: CD117
Late immature monocytes 1
Late immature monocytes 2
5.69
49.7
41.7
2.94
Late immature monocytes 4
Late immature monocytes 3
Comp-PE-A :: CD115
Comp-BV605-A :: Ly6C
Mature Ly6Chi monocytes
88.0
Mature Ly6Clo monocytes
9.05
Comp-BV605-A :: Ly6C
Comp-PE-A :: CD115

Supplementary Figure 6: Gating strategy used to identify populations from the murine bone marrow. Quality control gates, including time, single cells, non-debris and live cell gates were applied before analysing cells. In the infected BM Sca1 is substantially upregulated, disenabling the gating out of Sca1+ progenitors and stem cells.

Supp 7

Mock-infected bone marrow - Cell # and %

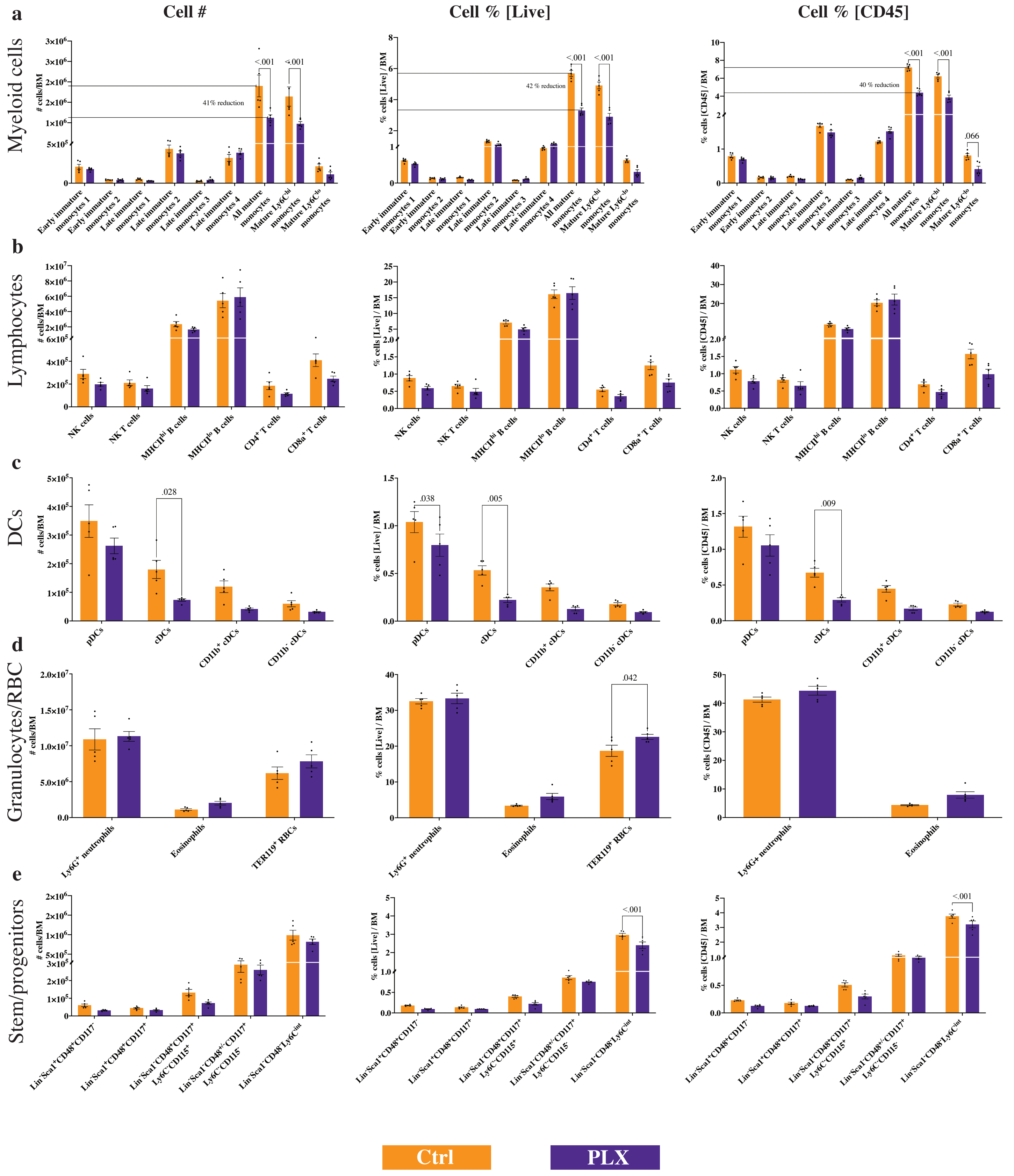

Supplementary Figure 7: Changes in CD45+ cell subsets in the bone marrow of mock-infected mice feed PLX5622. (a-e) Number of cells (left panel), percent out of total live cells, (middle panel) and percent out of total CD45+ cells (right panel) of myeloid cells (a), lymphocytes (b), DCs (c), granulocytes and RBCs (d) and stem/progenitors cells (e) in mock-infected PLX and mock-infected Ctrl mice. Data is presented as mean ± SEM from one independent experiment with at least five mice per group. p values were determined using a Two-way ANOVA and a Tukey's multiple comparisons test.

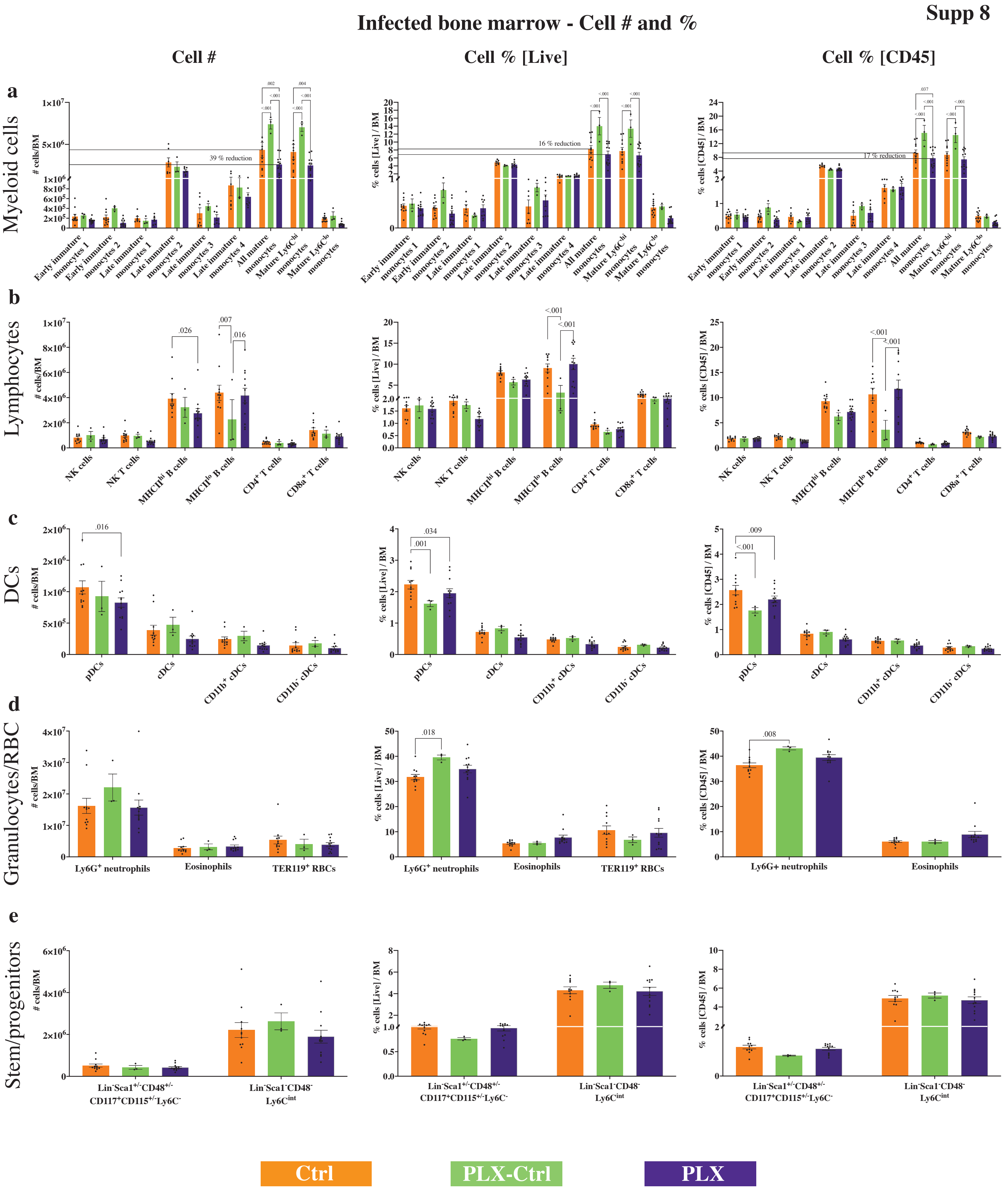

Supplementary Figure 8: Changes in CD45+ cell subsets in the bone marrow of infected mice feed PLX5622. (a-e) Number of cells (left panel), percent out of total live cells, (middle panel) and percent out of total CD45+ cells (right panel) of myeloid cells (a), lymphocytes (b), DCs (c), granulocytes and RBCs (d) and stem/progenitors cells (e) in Ctrl, PLX-Ctrl and PLX mice at dpi 7. Data is presented as mean ± SEM from one-three independent experiment with at least three mice per group. p values were determined using a Two-way ANOVA and a Tukey's multiple comparisons test.

Mock-infected bone marrow

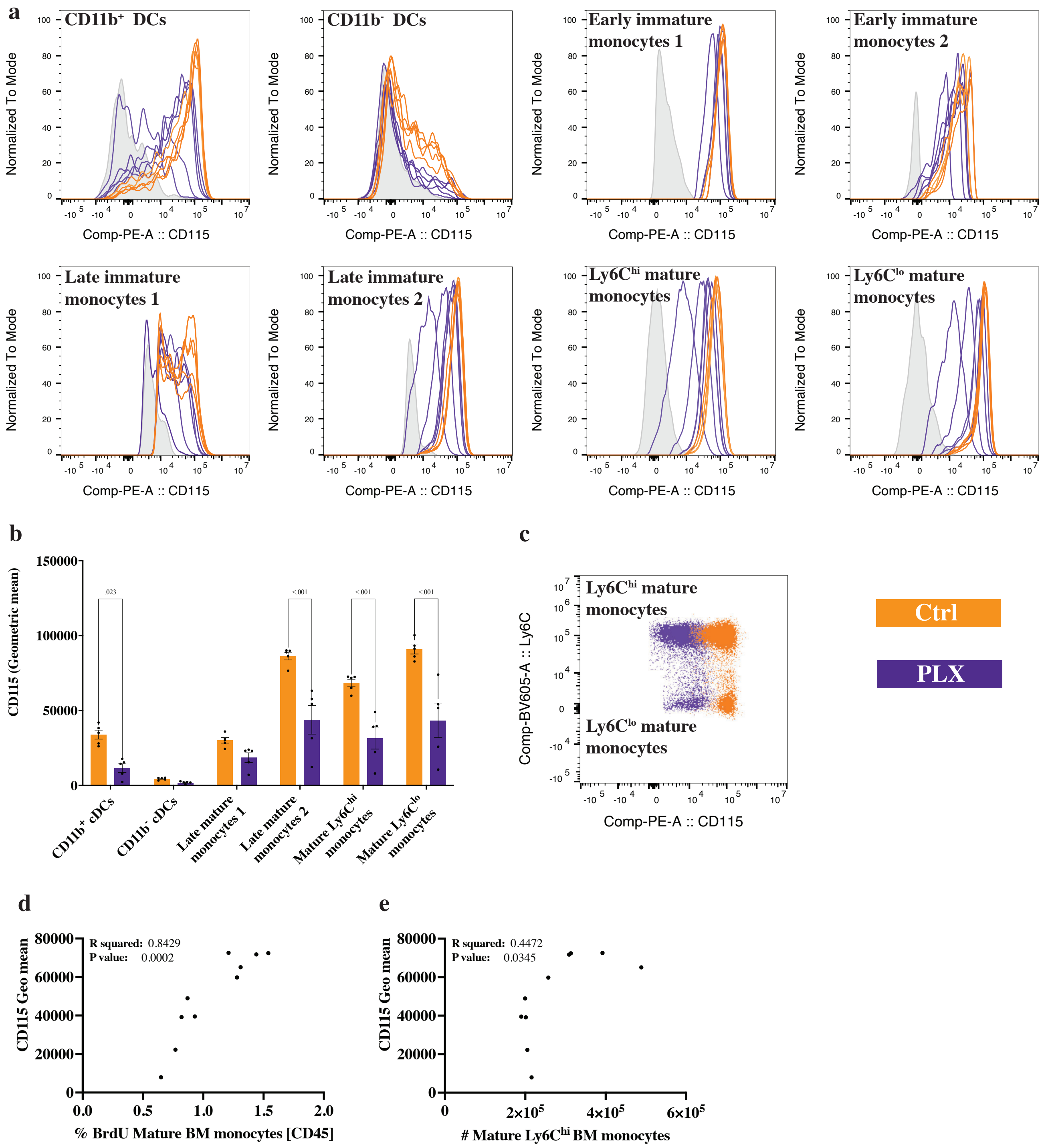

Supplementary Figure 9: CD115 is downregulated on DCs and monocytes in the bone marrow of mice treated with PLX5622. (a, b) Histograms (a) and bar graph (b) showing the expression of CD115 on DCs and monocytes in mock-infected PLX and mock-infected Ctrl mice. (c) Dot plot showing the reduced expression of CD115 on Ly6Chi and Ly6Clo mature BM monocytes in PLX5622-treated mice, relative to control chow feed mice. (d) Correlation analysis between the expression of CD115 on Ly6Chi mature BM monocytes and the frequency of BrdU+ Ly6Chi mature BM monocytes out of CD45+ BM cells. (e) Correlation analysis between the expression of CD115 on Ly6Chi mature BM monocytes and the number of Ly6Chi mature monocytes in the BM.

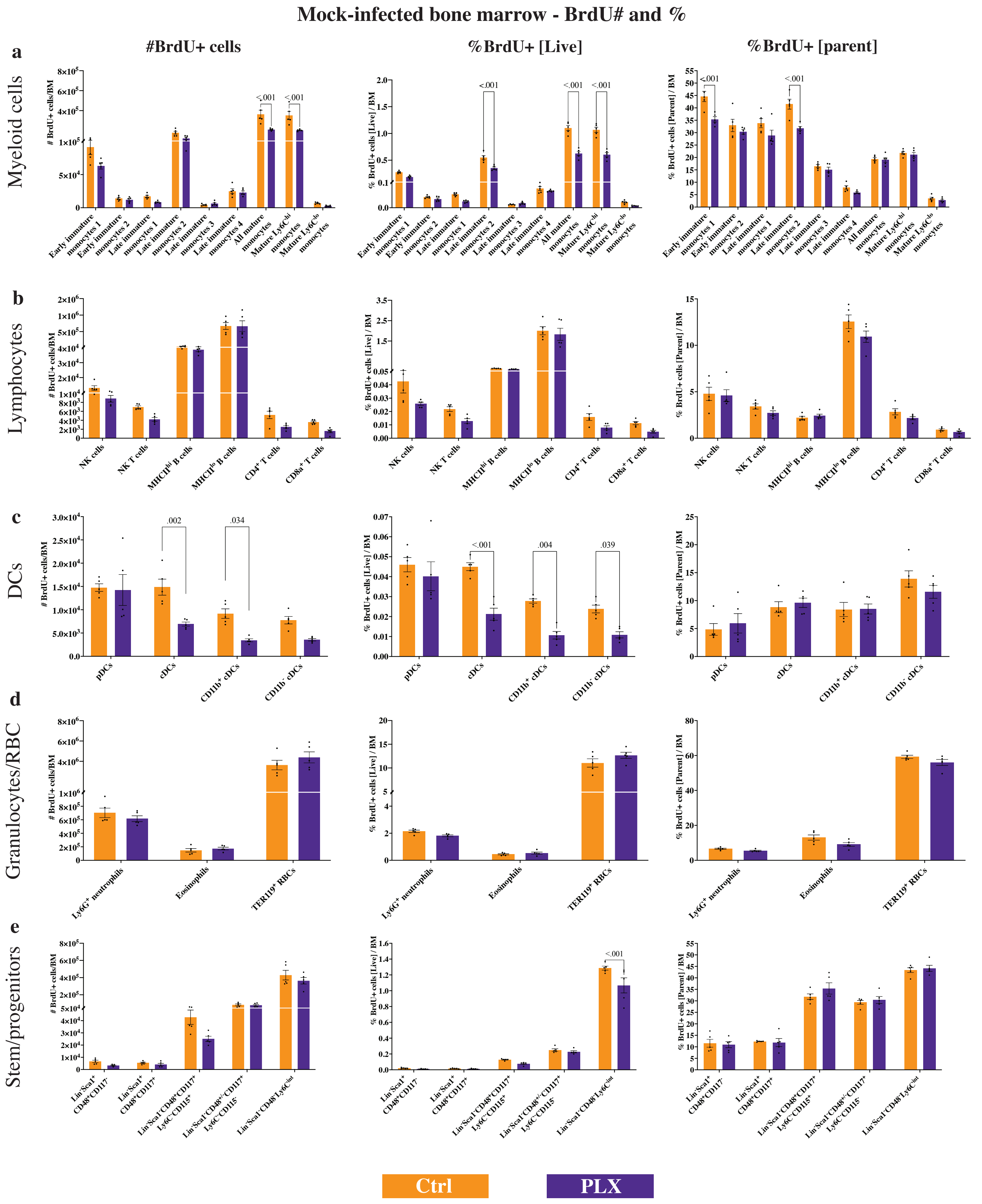

Supplementary Figure 10: Changes in the proliferative capacity of CD45+ cell subsets in bone marrow of mock-infected mice feed PLX5622. (a-e) Number and percent out of live and total population (i.e., parent gate) of BrdU+ myeloid cells (a), lymphocytes (b), DCs (c), granulocytes and RBCs (d) and stem/progenitors cells (e) in mock-infected PLX and mock-infected Ctrl mice. Data is presented as mean ± SEM from one independent experiment with at least five mice per group. p values were determined using a Two-way ANOVA and a Tukey's multiple comparisons test.

Infected bone marrow - BrdU# and %

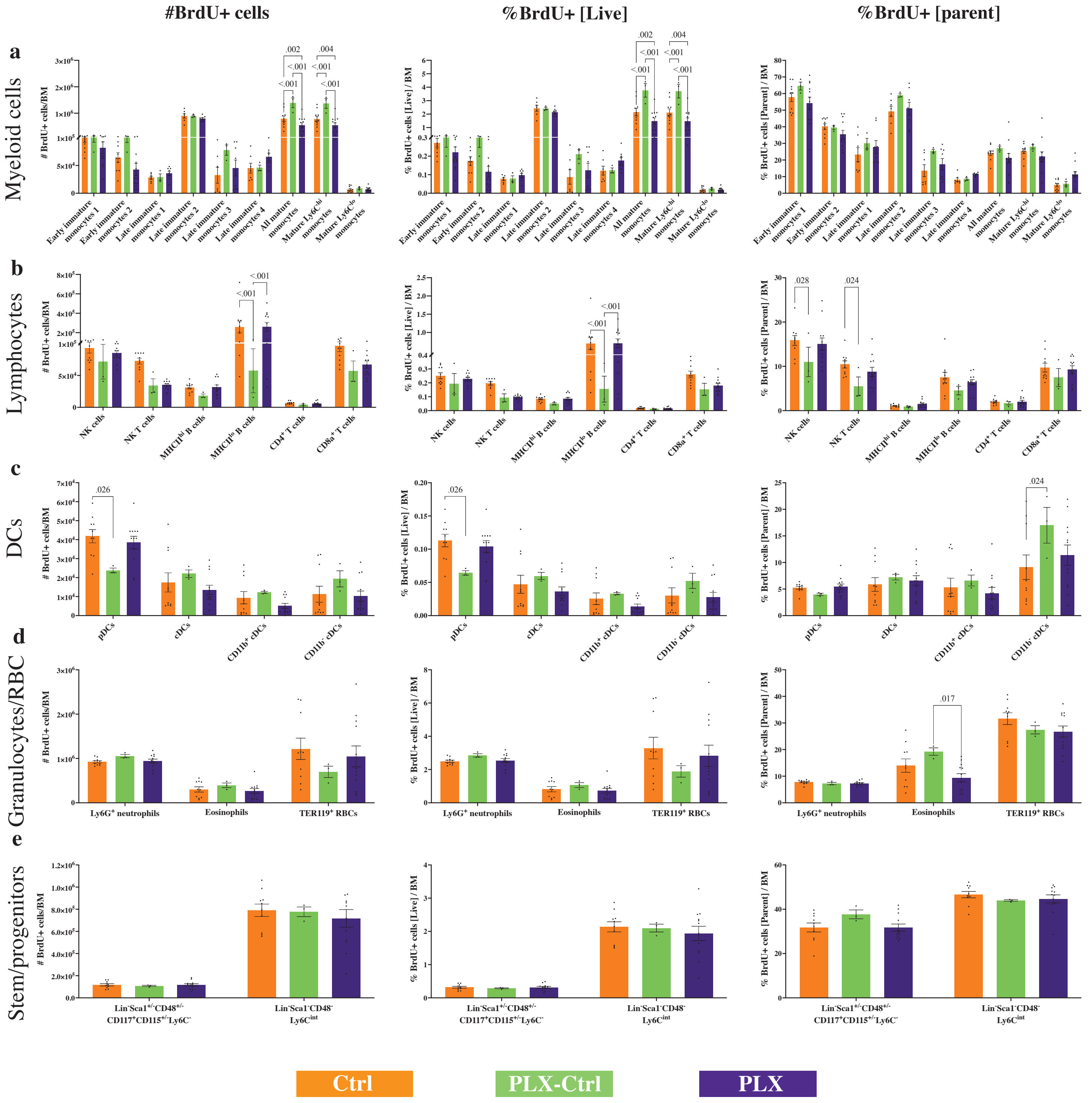

Supplementary Figure 11: Changes in the proliferative capacity of CD45+ cell subsets in bone marrow of infected mice feed PLX5622. (a-e) Number and percent out of live and total population (i.e., parent gate) of BrdU+ myeloid cells (a), lymphocytes (b), DCs (c), granulocytes and RBCs (d) and stem/progenitors cells (e) in Ctrl, PLX-Ctrl and PLX mice at dpi 7. Data is presented as mean ± SEM from one-three independent experiment with at least three mice per group. p values were determined using a Two-way ANOVA and a Tukey's multiple comparisons test.

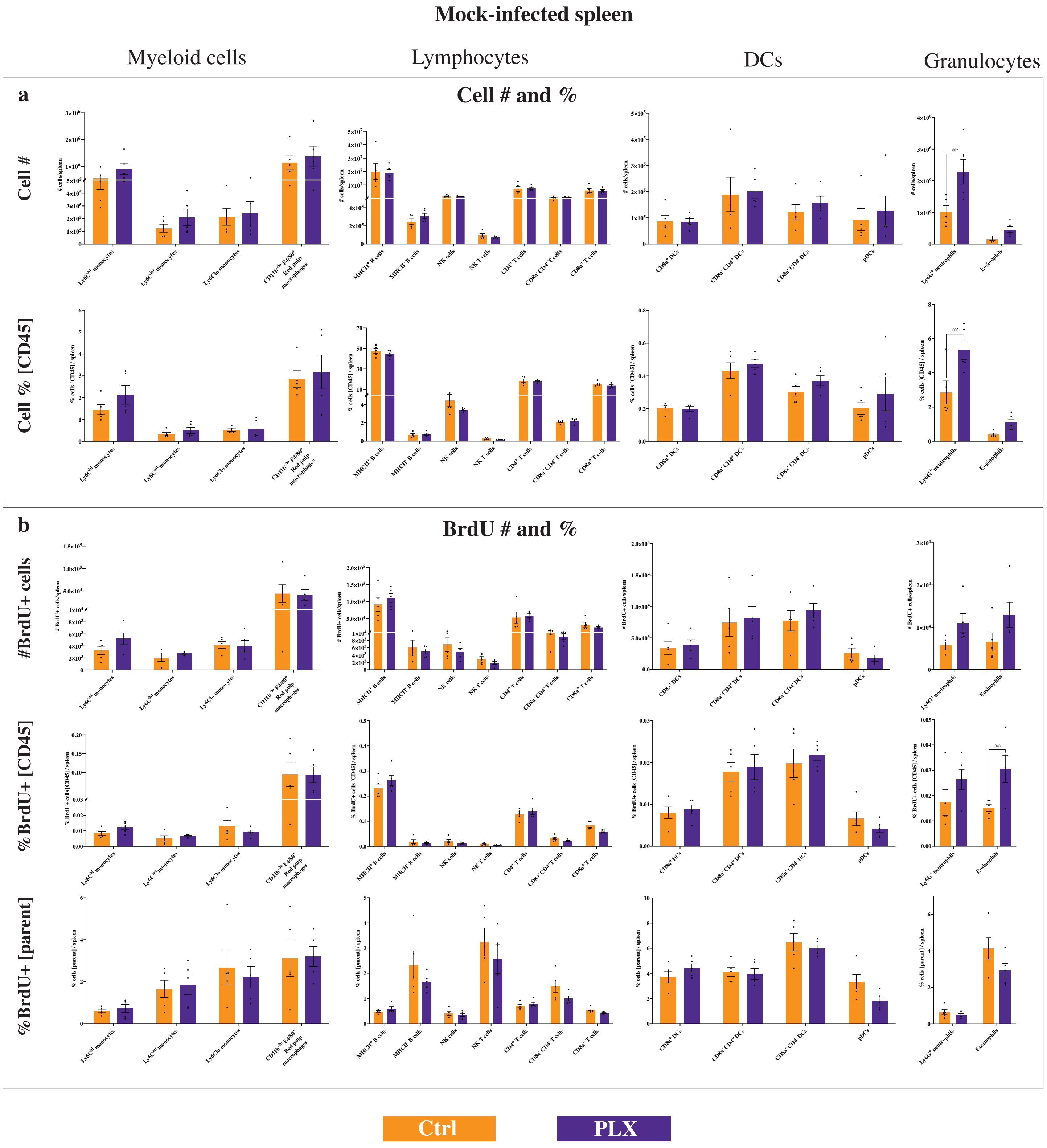

Supplementary Figure 12: Changes in CD45+ cell subsets from the spleen of mock-infected mice treated with PLX5622. (a) Number and percent out of live and total CD45+ cells of myeloid cells, lymphocytes, DCs and granulocytes in mock-infected PLX and mock-infected Ctrl mice. (b) Number and percent out of live and total population (i.e., parent gate) of BrdU+ proliferating myeloid cells, lymphocytes, DCs and granulocytes in mock-infected PLX and mock-infected Ctrl mice. Data is presented as mean ± SEM from one independent experiment with at least five mice per group. p values were determined using a Two-way ANOVA and a Tukey's multiple comparisons test.

Supplementary table 1: Criteria used to determine disease scores

Score	Criteria
0	No evidence of disease
1	Weight loss ≤ 3%
2	Weight loss ≤ 4%
3	Weight loss ≤ 5%
4	Weight loss ≥ 5%, reduced and slow movement and weak when scuffed.<br>**Add 0.1** for each disease sign: ruffled fur, hyperemic face, hunched posture or shaking<br>**Add 0.2** for each disease sign: no reaction upon pulling tail or stumbles when walks
4.9	Weight loss ≥ 5% and incapacitated (lying on side)

Supplementary table 2: Primer sequences used for qPCR

Primer	NM	Primer set sequence F 5'-3'	Primer set sequence R 5'-3'
Tnfa	NM_013693.3	ATGGCCTCCCTCTCATCAGT	GTTTGCTACGACGTGGGCTA
Il-1a	NM_010554.4	CATAACCCATGATCTGGAAG	ATTCATGACAAACTTCTGCC
Ccl2	NM_011333.3	CAAGATGATCCCAATGAGTAG	TTGGTGACAAAAACTACAGC
Ccl3	NM_011337	CCATATGGAGCTGACACCCC	GAGCAAAGGCTGCTGGTTTC
Ccl5	NM_013653	TGCTCCAATCTTGCAGTCGT	GCAAGCAATGACAGGGAAGC
Cxcl16	NM_023158.6	CCATTCTTTATCAGGTTCCAG	CTTGAGGCAAATGTTTTTGG

Supplementary table 3: Antibodies used for flow cytometry

Antibody	Clone	Company
Surface antibodies		
anti-CD11b	M1/70	Biolegend and BD Biosciences, USA
anti-CD11c	HL3	Biolegend
Anti-CD11c	N418	BD Biosciences, USA
anti-B220	RA3-6B2	Biolegend and BD Biosciences, USA
anti-CD8a	53-6.7	BD Biosciences, USA
anti-CX3CR1	SA011F11	Biolegend, USA
anti-CD117	2B8	Biolegend, USA
anti-I-A/I-E	M5/114.15.2	Biolegend, USA
anti-Ly6C	HK1.4	Biolegend, USA
anti-Ly6G	1A8	Biolegend, USA
anti-F4/80	BM8	Biolegend, USA
anti-CD4	RM4-5	Biolegend, USA
anti-Sca-1	D7	Biolegend, USA
anti-P2RY12	S16007D	Biolegend, USA
anti-CD64	X54-5/7.1	Biolegend, USA
anti-Siglec-H	551	Biolegend, USA
anti-TER119	TER-119	BD Biosciences, USA
anti-CD45	30-F11	BD Biosciences, USA
anti-CD3ε	145-2C11	Biolegend and BD Biosciences, USA
anti-NK1.1	PK136	BD Biosciences, USA
anti-CD115	AFS98	Biolegend, USA
anti-CD86	GL-1	Biolegend, USA
anti-CD48	HM48-1	Biolegend, USA
Intracellular antibodies		

Anti-CD68	FA-11	Biolegend, USA
anti-CD206	C068C2	Biolegend, USA

Supplementary table 4: Phenotypic markers used to identify myeloid subsets in the BM

Cell gated	Phenotype - non-infected BM	*Phenotype - infected BM
**Early immature monocytes**	**$CD45^{+}$, Lin^{-}, $CD11c^{lo}$, $CD117^{+}$, $Sca1^{-}$, $CD48^{+}$, $CD11b^{-}$**	**$CD45^{+}$, Lin^{-}, $CD11c^{lo}$, $CD117^{+}$, $Sca1^{+}$, $CD48^{+}$, $CD11b^{-}$**
Early immature monocytes 1	$CD115^{hi,}$ $Ly6C^{hi}$	$CD115^{hi}$, $Ly6C^{hi}$
Early immature monocytes 2	$CD115^{int}$, $Ly6C^{int}$	$CD115^{int}$, $Ly6C^{int}$
**Late immature monocytes**	**$CD45^{+}$, $Lin^{-,}$ $CD11c^{lo,}$ $CD117^{-}$, $Sca1^{-}$, $CD48^{+}$, $CD11b^{-}$**	**$CD45^{+}$, Lin^{-}, $CD11c^{lo}$, $CD117^{-}$, $Sca1^{+}$, $CD48^{+}$, $CD11b^{-}$**
Late immature monocytes 1	$Ly6C^{-}$, $CD115^{+}$	$Ly6C^{-}$, $CD115^{+}$
Late immature monocytes 2	$Ly6C^{+}$, $CD115^{+}$	$Ly6C^{+}$, $CD115^{+}$
Late immature monocytes 3	$Ly6C^{+}$, $CD115^{-}$	$Ly6C^{+}$, $CD115^{-}$
Late immature monocytes 4	$Ly6C^{-}$, $CD115^{-}$	$Ly6C^{-}$, $CD115^{-}$
**Mature monocytes**	**$CD45^{+}$, Lin^{-}, $CD11c^{lo}$, $CD117^{-}$, $Sca1^{-}$, $CD48^{+}$, $CD11b^{+}$, $CD115^{+}$**	**$CD45^{+}$, Lin^{-}, $CD11c^{lo}$, $CD117^{-,}$ $Sca1^{+}$, $CD48^{+}$, $CD11b^{+}$, $CD115^{+}$**
Mature Ly6Chi monocytes	$Ly6C^{hi}$	$Ly6C^{hi}$
Mature Ly6Clo monocytes	$Ly6C^{lo}$	$Ly6C^{lo}$

* Cells in the infected BM upregulate Sca1
